# Mechanical Activation of cPLA2 Impedes Fatty Acid β‐Oxidation in Vein Grafts

**DOI:** 10.1002/advs.202411559

**Published:** 2024-11-26

**Authors:** Linwei Fan, Yuanjun Tang, Jian Liu, Yueqi Liu, Yiwei Xu, Jiayu Liu, Han Liu, Wei Pang, Yuxuan Guo, Weijuan Yao, Tao Zhang, Qin Peng, Jing Zhou

**Affiliations:** ^1^ Department of Physiology and Pathophysiology School of Basic Medical Sciences State Key Laboratory of Vascular Homeostasis and Remodeling Department of Cardiology and Institute of Vascular Medicine Peking University Third Hospital National Health Commission Key Laboratory of Cardiovascular Molecular Biology and Regulatory Peptides Beijing Key Laboratory of Cardiovascular Receptors Research Peking University Beijing 100191 China; ^2^ Shenzhen Bay Laboratory Shenzhen 518132 China; ^3^ Institute of Cardiovascular Sciences School of Basic Medical Sciences State Key Laboratory of Vascular Homeostasis and Remodeling Beijing Key Laboratory of Cardiovascular Receptors Research Peking University Beijing 100191 China; ^4^ Department of Vascular Surgery Peking University People's Hospital Beijing 100044 China

**Keywords:** cell proliferation and migration, fatty acid β‐oxidation, mechanical force, mechanosensory, vascular smooth muscle cell

## Abstract

High‐magnitude cyclic stretch from arterial blood pressure significantly contributes to the excessive proliferation and migration of vascular smooth muscle cells (VSMCs), leading to neointima formation in vein grafts. However, the molecular mechanisms remain unclear. This study highlights the critical role of cytosolic Phospholipase A2 (cPLA2)/ Yin Yang 1 (YY1)/ carnitine palmitoyltransferase 1b (CPT1B) signaling in coordinating VSMC mechanical activation by inhibiting fatty acid β‐oxidation. Metabolomic analysis showed that a 15%–1 Hz arterial cyclic stretch, compared to a 5%–1 Hz venous stretch, increased long‐chain fatty acids in VSMCs. cPLA2, identified as a mechanoresponsive molecule, produces excessive arachidonic acid (ArAc) under the 15%–1 Hz stretch, inhibiting CPT1B expression, a key enzyme in fatty acid β‐oxidation. ArAc promotes transcription factor YY1 degradation, downregulating CPT1B. Inadequate fatty acid oxidation caused by knockdown of CPT1B or YY1, or etomoxir treatment, increased nuclear membrane tension, orchestrating the activation of cPLA2. Overexpressing CPT1B or inhibiting cPLA2 reduced VSMC proliferation and migration in vein grafts, decreasing neointimal hyperplasia. This study uncovers a novel mechanism in lipid metabolic reprogramming in vein grafts, suggesting a new therapeutic target for vein graft hyperplasia.

## Graphical abstract

The mechanosensory protein cytosolic Phospholipase A2 (cPLA2) is activated by arterial stretch, leading to the ubiquitination of transcription factor Yin Yang 1 (YY1), which in turn mediates the downregulation of carnitine palmitoyltransferase 1b (CPT1B). As a result, fatty acid β‐oxidation (FAO) is impaired which leads to the proliferation and migration of vascular smooth muscle cells (VSCMs) by upregulating reactive oxygen species (ROS) levels. Moreover, Impaired FAO can further increase nuclear membrane tension, thereby orchestrating the activation of cPLA2.

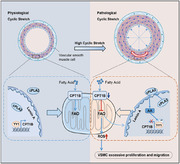

## 1 Introduction

Coronary artery bypass graft (CABG) surgery is the gold standard treatment for patients with obstructive coronary artery disease.^[^ ^1^ ^]^ The most commonly used conduit in CABG surgery is saphenous vein graft (VG), which fails in 40–50% of treated patients within 10 years after surgery due to graft occlusion.^[^ ^2^ ^]^ VG stenosis and occlusion are characterized by neointimal hyperplasia within 1–12 months after grafting, eventually progressing to atherosclerosis in the late stages.^[^ ^3^, ^4^ ^]^ These processes are driven by complex pathophysiological factors, including altered hemodynamic forces after vein grafting.^[^ ^5^ ^]^ The exposure of VGs to increased arterial pressure (from 0–30 to ≈150 mmHg) is a significant determinant of obliterative neointimal hyperplasia.^[^ ^6^, ^7^ ^]^ Mechanistically, the subjection of VG vascular smooth muscle cells (VSMCs) to high‐magnitude cyclic stretch has been shown to promote VSMCs proliferation and migration, leading to adverse vascular remodeling.^[^ ^8^, ^9^ ^]^ However, the key molecules and pathways involved in the mechanical regulation of VSMC function and behavior remain poorly understood.

There is evidence that cyclic stretch affects the biological behavior of cells by inducing metabolic reprogramming,^[^ ^10^, ^11^ ^]^ including our previous study demonstrating that arterial stretch enhances the transition from oxidative phosphorylation to glycolysis to promote VSMC proliferation and migration.^[^ ^11^ ^]^ Currently, despite sporadic reports suggesting that changes in VSMC metabolism may accompany the occurrence of vascular diseases,^[^ ^12^, ^13^, ^14^ ^]^ the metabolic characteristics of VSMC and its regulatory factors under physiological and pathological conditions are still a mystery. Among various metabolic pathways, fatty acid β‐oxidation (FAO) is the most efficient way to degrade long‐chain fatty acids to generate energy.^[^ ^15^ ^]^ In the cytosol, long‐chain fatty acids are first activated to form fatty acyl‐coenzyme A (fatty acyl‐CoA). Fatty acyl‐CoA is then transported from the cytosol into the mitochondria via carnitine palmitoyltransferase 1 (CPT1) located in the mitochondrial outer membrane. Subsequently, fatty acyl‐CoA undergoes FAO to produce acetyl‐CoA, which can enter the citric acid cycle.^[^ ^15^ ^]^ Impairment of mitochondrial FAO therefore leads to the accumulation of long‐chain free fatty acids in the cytosol, which mediate lipotoxicity. Accumulation of lipids affect the cell behaviors such as cell proliferation and migration via multiple mechanisms, including activation of endoplasmic reticulum stress, modification of mitochondrial function and oxidative stress.^[^ ^16^, ^17^, ^18^ ^]^ Functionally, inhibition of FAO by inactivating the CPT1 isoform carnitine palmitoyltransferase 1b (CPT1B) stimulated cardiomyocyte proliferation and enabled heart regeneration in mice.^[^ ^19^ ^]^ Increasing serum levels of long‐chain fatty acids (e.g., palmitate) in mice induced VSMC proliferation and inflammation, thereby promoting neointimal formation after vascular injury.^[^ ^20^ ^]^ Although FAO is suggested to be involved in the functional modulation of VSMCs,^[^ ^16^, ^21^, ^22^ ^]^ the exact role of FAO and how it is regulated in neointimal hyperplasia remain unknown.

Cells are capable of sensing and responding to various mechanical signals in their microenvironment, such as mechanical stretch, matrix stiffness, geometric constraints, and osmotic pressure. Emerging evidence has revealed a direct role of the nucleus in cellular mechanosensing and mechanotransduction.^[^ ^23^, ^24^ ^]^ Extracellular mechanical forces can be transmitted to the nucleus through the cytoskeleton, including actin microfilaments and microtubules, and the linker of nucleoskeleton and cytoskeleton (LINC) complex.^[^ ^25^ ^]^ This transmission leads to the activation of nuclear mechanosensory structures or molecules, such as the cytosolic Phospholipase A2 (cPLA2).^[^ ^26^ ^]^ cPLA2 is a calcium‐dependent phospholipase that preferentially hydrolyzes phospholipids containing arachidonic acid (ArAc), playing a role in lipid metabolism.^[^ ^27^ ^]^ Study has shown that the activation of cPLA2 during nuclear swelling is a mechanical conduction process mediated by nuclear membrane tension.^[^ ^28^ ^]^ The permeable expansion of the nucleus results in the transfer of cPLA2 from the nucleoplasm to the nuclear envelope.^[^ ^28^, ^29^ ^]^ However, the mechanosensory role of cPLA2 in mediating VSMC responses to mechanical stretch has not yet been reported.

Our current study identified a suppression of FAO in VSMCs during VG neointima formation induced by arterial cyclic stretch. Our findings revealed that vein grafting and 15%‐1 Hz arterial cyclic stretch downregulated the expression of CPT1B, impeded FAO, and increased the content of long‐chain fatty acids, the substrate of CPT1B, in VGs or stimulated VSMCs. Mechanistic studies demonstrated that cyclic stretch activated the mechanosensory cPLA2, generating ArAc which mediates a ubiquitin‐protease‐dependent degradation of Yin Yang 1 (YY1), leading to the transcriptional inhibition of CPT1B. Additionally, we discovered that a deficiency in YY1 or CPT1B increased nuclear membrane tension, further orchestrating cPLA2 activation. Targeting the cPLA2/YY1/CPT1B signaling cascade by inhibiting cPLA2 activation or overexpressing CPT1B reduced fatty acid accumulation, cell proliferation and migration, and post‐grafting neointimal hyperplasia. Based on these results, we propose that the cPLA2/YY1/CPT1B axis plays a crucial role in VSMC responses to mechanical forces and vein graft neointimal hyperplasia. Our findings also suggest that therapeutically targeting this axis could be a viable strategy for preventing or treating vein graft failure.

## 2 Results

### 2.1 FAO and CPT1B Expression are Suppressed in VSMCs within VGs

**Figure 1:**
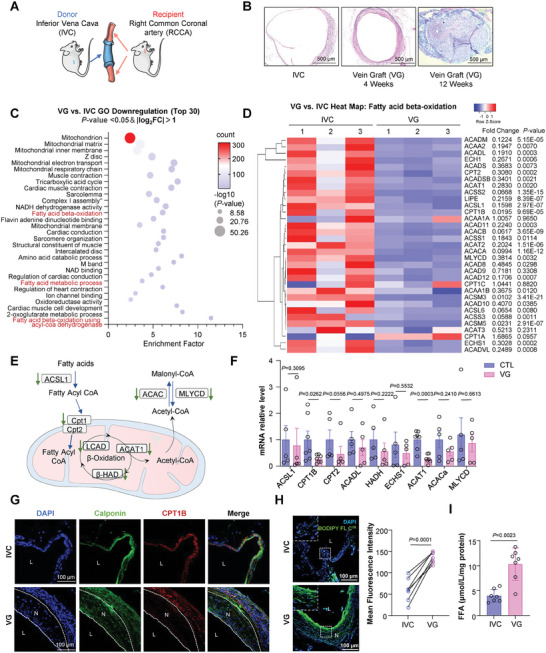
Fatty acid β‐oxidation (FAO) and CPT1B expression are suppressed in VSMCs within VGs. A) Schematic representation of the mouse vein graft (VG) model. B) Representative H&E staining of cross sections of the inferior vena cava (IVCs) and VGs at 4 and 12‐ weeks post‐surgery. C) Gene ontology (GO) analysis of genes significantly decreased in VGs compared to IVCs (*p‐*value <0.05 and |log_2_ FC|>1). The X‐axis represents the gene enrichment factor and the Y‐axis represents the top 30 most significantly regulated GO terms. The dot size indicates the ‐log10(*p*‐value) and the dot color indicates gene counts enriched in the pathway. D) Heatmap showing the expression of genes related to FAO in VGs versus IVCs. The scale bar ranges from −1 to 1, with blue indicating lower expression and red indicating higher expression. Specific fold changes and P‐values are marked on the right side of the heatmap. E) Schematic illustration of the mitochondrial FAO pathway, highlighting key enzymes with downregulated expression as indicated in panel (D) by arrows. F) Quantitative RT‐qPCR validation of key FAO‐related genes in IVCs and 6‐week VGs. *n* = 7 biological replicates. Data were analyzed using a parametric unpaired *t*‐test. G) Representative immunofluorescent images of CPT1B in IVCs and 6‐week VGs. Vascular smooth muscle cells (VSMCs) are indicated by Calponin‐positive signals. L: lumen; N: neointima. H) Representative BODIPY FL C16 fluorescent images of IVCs and 6‐week VGs. Mean fluorescence intensity was quantified using ImageJ software; *n* = 7 biological replicates. I) Free fatty acid (FFA) content in 6‐week VGs and autologous control IVCs, normalized to tissue total protein concentration and expressed as µmol/L/mg protein. *n* = 7 biological replicates. Data were analyzed using unpaired (F) or paired (I and H) Student *t*‐test. Error bars show ± SEM. Exact *p*‐values between the indicated groups are presented.

We utilized a mouse model of vein grafting using a “cuff” technique,^[^ ^30^ ^]^ analogous to human CABG surgery. The inferior vena cava (IVC) from donor mice was harvested and grafted into the carotid artery of the recipient by placing the venous ends over an arterial cuff and suturing them together (**Figure** **1**A). Blood flow in the grafted vein was measured by ultrasound to assess the patency of the vascular access (Figure S1, Supporting Information), and mice with graft occlusion were excluded for further analysis. Four weeks post‐surgery, neointimal hyperplasia developed in VGs, causing lumen narrowing; after 12 weeks, excessive neointimal hyperplasia led to vascular obstruction (Figure 1B), indicative of the successful establishment of the model. To investigate the impact of vein grafting on gene expression, we analyzed our previous RNA‐sequencing data from VGs and autologous control IVCs 4 weeks post‐transplantation in mice.^[^ ^11^ ^]^ Gene ontology analysis of the differentially expressed genes revealed that biological pathways such as FAO, fatty acid metabolic process, and FAO using acyl‐CoA dehydrogenase were among the top 30 downregulated gene ontology terms in VGs compared to IVCs (Figure 1C; Figure S2A, Supporting Information). The heatmap displaying the expression of genes enriched in the FAO pathway showed downregulation of a serial of gene encoding enzymes (Figure 1D), most of which play key roles in mitochondrial FAO (Figure 1E). This finding suggests a potential inhibition of FAO metabolism in VGs. To explore the clinical relevance of this finding, we examined Martinez et al.’s RNA‐sequencing dataset of human arteriovenous fistulae (AVF),^[^ ^31^ ^]^ as the creation of both VGs and AVF involves the adaption of veins to the arterial environment.^[^ ^32^ ^]^ In Martinez's study, RNA expression profiles were obtained from 19 native veins and 19 AVF samples from 64 patients undergoing 2‐stage AVF surgeries at a single center. Gene ontology (the top 20) and heatmap analysis (the top 30) of differentially expressed genes from the both 11 mature and failed AVFs versus 10 native veins revealed a pathway responsible for the positive regulation of lipid metabolic process and downregulation of genes encoding enzymes, such as CPT1B, involved in FAO in AVF (Figure S2B–D, Supporting Information).

CPT1 is the rate‐limiting enzyme responsible for shuttling fatty acyl‐CoA into mitochondria for FAO. There are three known isoforms of CPT1: CPT1A, CPT1B, and CPT1C, each encoded by different genes and differentially expressed depending on tissue and cell type.^[^ ^33^ ^]^ Quantitative RT‐PCR analysis, which confirmed the RNA‐sequencing data, demonstrated significantly reduced mRNA levels of FAO‐related genes, particularly CPT1B and ACAT1 (Figure 1F). Furthermore, immunofluorescence showed lower expression of CPT1B protein in intimal layer of VGs, compared to the media layer of VGs or IVCs (Figure 1G). To further investigate whether vascular lipid metabolism is impaired after vein grafting, we used BODIPY FL C16, a fluorescent palmitate analogue, for in vivo imaging of fatty acid uptake and metabolism. Each mouse received a tail vein injection of 100 µL of 200 µmol L^−1^ BODIPY FL C16. Six hours post‐injection, VGs and autologous control IVCs were harvested for frozen sectioning. Fluorescent imaging indicated excessive accumulation of BODIPY FL C16 in VSMCs of mouse VGs compared to IVCs (Figure 1H). Enzyme‐linked immunosorbent assay determined the long‐chain free fatty acid (FFA) content in the vascular tissue homogenate, revealing significantly higher FFA content in VGs compared to IVCs (Figure 1I). Taken together, these results demonstrated that FAO and CPT1B expression are suppressed in VSMCs within VGs, indicating impaired vascular lipid metabolism following vein grafting.

### 2.2 High‐Magnitude Cyclic Stretch Impedes FAO to Promote VSMC Proliferation and Migration

**Figure 2:**
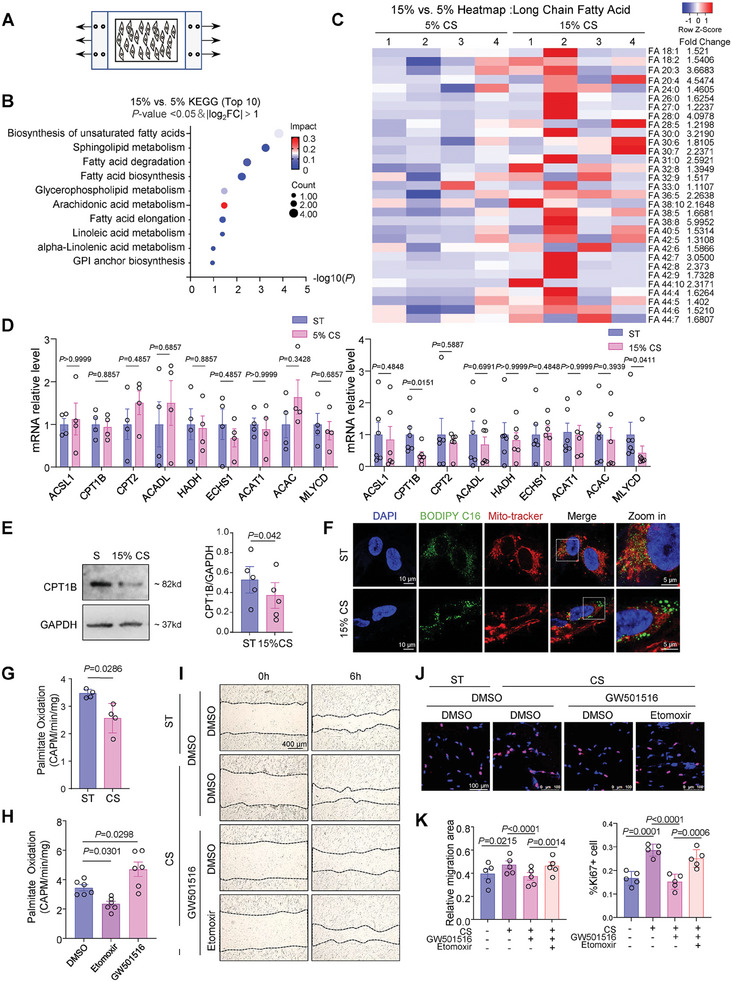
High‐magnitude cyclic stretch impedes FAO to promote VSMC proliferation and migration. A) Schematic representation of the cell cyclic stretch (CS) experiment. B) KEGG analysis of lipid metabolites significantly decreased in VSMCs under 15% compared to 5% CS for 24 h (*p*‐value <0.05 and |log_2_ FC|>1). The X‐axis represents the ‐log10 (*p*‐value), and the Y‐axis shows the top 10 most significantly regulated KEGG terms. Dot size indicates the impact, while dot color indicates counts enriched in the pathway. C) Heatmap showing the significant decrease in long‐chain fatty acid contents in cells under 15% CS versus 5% CS. The heatmap scale ranges from ‐1 to 1, with red indicating higher metabolite content. D) Quantitative RT‐qPCR validation of FAO‐related gene expression in VSMCs subjected to 5% CS or 15% CS relative to static conditions (ST) for 12 h. *n* = 5 biological replicates (5% CS), *n* = 6 biological replicates (15% CS). E) Western blotting analysis and quantification of CPT1B protein levels in VSMCs under 15% CS or ST for 24 h. *n* = 5 biological replicates. F) Representative BODIPY FL C16 fluorescence images of VSMCs upon 15% CS or ST for 24 h. G) H^3^‐palmitate radioisotope experiment analyzing palmitate oxidation in VSMCs under 15% CS or ST for 24 h. *n* = 4 biological replicates. H) H^3^‐palmitate radioisotope experiment analyzing palmitate oxidation in VSMCs treated with CPT1 inhibitor etomoxir (5 µmol L) or PPAR receptor agonist GW501516 (10 µmol L^−1^) for 24 h. *n* = 6 biological replicates. I–K) Cell proliferation (I) and migration (J) in VSMCs treated with etomoxir, GW501516 or the combination of etomoxir and GW501516 upon 15% CS or ST for 24 h. Migration areas and the Ki67‐positive cell ratio were analyzed using ImageJ software (K). *n* = 5 biological replicates. Data were analyzed using unpaired Student *t*‐test (D and E), unpaired Mann‐Whitney test (G) or Two‐Way ANOVA (H and K). Error bars show ± SEM. Exact *p*‐values between the indicated groups are presented.

Next, we assessed the impact of arterial mechanical environment on cellular fatty acid metabolism by conducting a cyclic stretching experiment on cultured VSMCs (**Figure** **2**A). We applied uniaxial cyclic stretch with 15% cyclic strain and 1 Hz frequency to simulate arterial mechanical conditions, and uniaxial cyclic stretch with 5% cyclic strain and 1 Hz frequency was used to simulate venous mechanical conditions.^[^ ^34^, ^35^ ^]^ We performed untargeted ultra‐performance liquid chromatography coupled to mass spectrometry (UPLC‐MS)‐based lipidomics on VSMCs subjected to 15% or 5% stretch for 24 h. Partial least squares discriminant analysis plots revealed distinct metabolic signatures in cells undergoing 5% versus 15% cyclic stretch (Figure S3A, Supporting Information). Heatmap analyses of hierarchical clustering of differentially abundant metabolites showed that lipid levels in VSMCs under 15% stretch were significantly increased compared to those under 5% stretch (Figure S3B, Supporting Information). KEGG pathway analysis indicated that pathways such as fatty acid degradation were affected (Figure 2B). The table (Table S1, Supporting Information) and heatmap displaying fatty acids, specifically the significantly different (*P *< 0.05) long chain fatty acids (Figure 2C) (i.e., fatty acids with more than 18 carbon atoms) indicated that their levels were higher in VSMCs under 15% stretch compared to 5% stretch, suggestive of impeded FAO in VSMCs under 15% stretch.

Quantitative RT‐PCR analysis showed no significant changes in the mRNA levels of FAO‐related genes previously tested in VGs and IVCs (Figure 1F) in VSMCs subjected to 5% cyclic stretch or kept in a static condition for 12 h (Figure 2D, left). This suggests that VSMCs maintain physiological levels of lipid metabolism under both 5% stretch and static conditions. Consequently, static conditions were used as the control in subsequent experiments. Furthermore, the data showed that the mRNA levels of CPT1B and MLYCD were significantly reduced in VSMCs under 15% stretch compared to static conditions (Figure 2D, right). Considering the quantitative RT‐PCR results from both mouse vessels and cultured cells, CPT1B expression was confirmed to be suppressed in VGs and cells with high‐magnitude cyclic stretch. Western blotting further confirmed a significant decrease in CPT1B protein levels under 15% stretch relative to static conditions for 24 h (Figure 2E).

To visualize lipid uptake and metabolism in cultured cell, VSMCs were incubated with a medium containing 1 µmol L^−1^ BODIPY FL C16 for 2 h, washed, and then incubated with culture media for another hour to allow lipid metabolism to progress. The results showed that BODIPY FL C16 was rarely and homogeneously distributed in VSMCs under static conditions; however, BODIPY FL C16 accumulated in the form of lipid droplets and did not colocalize with mitochondria in VSMCs under 15% stretch (Figure 2F), suggestive of impeded degradation of BODIPY FL C16. We then used a radioisotope method to quantify the level of FAO. The principle of the assay is that mitochondrial FAO converts [9,10‐^3^H]‐palmitic acid to ^3^H_2_O and ^3^H_2_O, which can be separated based on differential diffusion rates.^[^ ^36^ ^]^ The data indicated that 15% cyclic stretch significantly reduced palmitate oxidation activity compared to the static condition (Figure 2G).

To explore the role of FAO dysregulation in mediating VSMC proliferation and migration, the most crucial cellular behavioral changes during VG hyperplasia, we used etomoxir (a CPT1 inhibitor that blocks cellular FAO) and GW501516 (a peroxisome proliferator‐activated receptor agonist known to boost FAO) to treat cells. Etomoxir decreased, while GW501516 increased, palmitate oxidation activity in VSMCs (Figure 2H), confirming the reliability of FAO intervention. Functionally, the results of scratch assay and Ki67 staining showed that VSMCs migration and proliferation were promoted by 15% stretch, but this effect was attenuated by treatment with GW501516; the attenuation effects were further abolished by treatment with etomoxir (Figure 2I–K). FAO impairment is closely linked to the production of reactive oxygen species (ROS) that may cause excessive cell proliferation.^[^ ^37^, ^38^, ^39^ ^]^ ROS are generated from mitochondria, cytochrome P450, and xanthine oxidase.^[^ ^40^ ^]^ Mitochondrial glutathione (GSH) uses nicotinamide adenine dinucleotide phosphate (NADPH) to neutralize excess ROS.^[^ ^41^ ^]^ Total and mitochondrial ROS (mito‐ROS) levels were assessed using DCFH‐DA and MitoSOX Red fluorescence, respectively. An increase in both total ROS and mito‐ROS production was observed in VSMCs exposed to 15% stretch for 12 h. This effect was mitigated by the overexpression of CPT1B (Figure S4A,B, Supporting Information). CPT1 inhibition also increased the mito‐ROS levels (Figure S4C, Supporting Information). Moreover, cyclic stretch and FAO inhibition reduced the NADPH contents (Figure S4D,E, Supporting Information), that may contribute to the increase of mito‐ROS (Figure S4C, Supporting Information). The stretch‐induced decrease of NADPH was blocked by CPT1B overexpression (Figure S4D, Supporting Information). Ki67 staining showed that treatment with N‐acetyl cysteine (NAC), a ROS scavenger, suppressed the cell proliferation in response to FAO inhibition (Figure S4F, Supporting Information). Altogether, these results indicate that high‐amplitude cyclic stretch impeded FAO, generating excess ROS to promote VSMC proliferation and migration.

### 2.3 High‐Magnitude Cyclic Stretch Actives Nuclear Mechanosensor cPLA2

**Figure 3:**
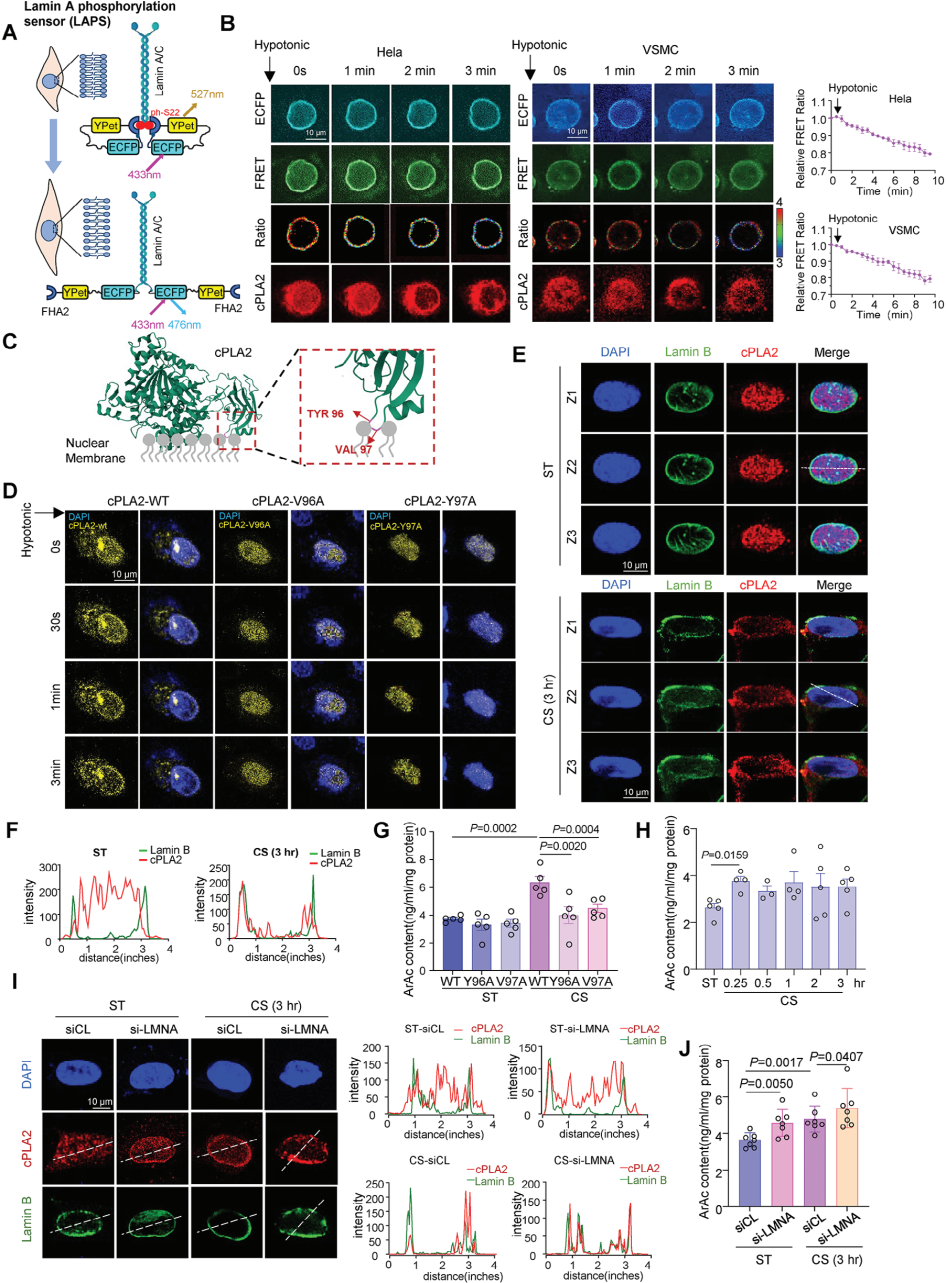
High‐magnitude cyclic stretch actives a nuclear mechanosensor cPLA2. A) Schematic diagram of the fluorescence resonance energy transfer (FRET)‐based Lamin‐A/C phosphorylation force sensor (LAPS). Low FRET efficiency indicates high nuclear membrane tension. B) Representative images of ECFP, FRET, FRET/ECFP ratio, and cPLA2‐mCherry in Hela cells and VSMCs transfected with LAPS and a cPLA2‐mCherry construct for 48 h, followed by hypotonic treatment. FRET ratios were calculated using the open‐source software Fluocell. C) Schematic diagram illustrating the insertion of the cPLA2 C2‐like domain into the nuclear envelope under stretch. D) Representative live cell images of cPLA2‐EYFP wildtype (WT) and mutants (V96A and Y97A) alongside Hoechst (nucleus) in VSMCs under hypotonic treatment. E) Representative immunofluorescent images of cPLA2 and nuclear envelope protein Lamin B1 in VSMCs subjected to 15% cyclic stretch (CS) or static (ST) conditions for 3 h. Z1‐Z3 indicate three different scanning layers. F) Fluorescence intensity profiling showing the distribution and overlap of cPLA2 and Lamin B from panel (E). G) Arachidonic acid (ArAc) production in VSMCs transfected with WT, V96A, and Y97A‐cPLA2 plasmids under 15% CS or ST conditions for 3 h. ArAc content normalized to total protein concentration and expressed as ng/mL/mg protein. *n* = 5 biological replicates. H) ArAc production in VSMCs under different 15% CS durations. *n* = 5 biological replicates. I) Representative immunofluorescent images of cPLA2 and Lamin B in VSMCs transfected with LMNA siRNA (si‐LMNA, 40 nmol L^−1^) or a control siRNA (siCL) and subjected to 15% CS or ST conditions for 3 h. Distribution of cPLA2 and Lamin B is quantified by fluorescence intensity profiling. J) ArAc Production in VSMCs treated as described in panel I). *n* = 7 biological replicates. Data were analyzed using unpaired Mann‐Whitney test (H) or Two‐Way ANOVA (G and J). Error bars show ± SEM. Exact *p*‐values between the indicated groups are presented.

We attempted to understand how VSMCs perceive and transduce cyclic stretch to downregulate CPT1B expression and inhibit FAO. cPLA2 has been identified as a nuclear mechanical sensor that detects dilation and tension in the nuclear membrane.^[^ ^28^ ^]^ Nuclear membrane tension can be visualized in real‐time using a fluorescence resonance energy transfer (FRET)‐based Lamin‐A/C phosphorylation sensor (LAPS)^[^ ^42^ ^]^ (**Figure** **3**A), which operates on the theory that serine‐22 phosphorylation of Lamin‐A/C induces nuclear envelope breakdown and decreases the nuclear membrane tension. We transfected the cells with LAPS and a cPLA2‐mCherry construct to simultaneously visualize nuclear membrane tension and cPLA2 activation, indicated by its translocation to the nuclear membrane. A lower FRET efficiency signaled higher nuclear membrane tension. To simulate mechanical stretch, we applied hypotonic conditions, which increased nuclear height and nuclear surface area (Figure S5A, Supporting Information). In wild‐type (WT) biosensors, the FRET ratio decreased under hypotonic stress (Figure S5B,C, Supporting Information), while an arginine‐605 to alanine (R605A) mutant LAPS showed no change (Figure S5D,E, Supporting Information), demonstrating the ability of the WT biosensor in measuring nuclear membrane tension. Both Hela cells and VSMCs showed increased nuclear membrane tension and cPLA2 translocation to the nuclear membrane under hypotonic treatment (Figure 3B), suggestive of cPLA2 activation. Stretching the lipid headgroups has been shown to promote the insertion of cPLA2 C2‐like domains and increase hydrophobic interaction between cPLA2 adsorption sites (e.g., tyrosine‐96 and valine‐97) (Figure 3C) and the nuclear membrane.^[^ ^43^ ^]^ We created cPLA2 mutants tyrosine‐96 and valine‐97 to alanine (Y96A and V97A) to decrease membrane affinity. Live cell fluorescence imaging revealed that these mutants suppressed cPLA2 activation under hypotonic stress, unlike WT cPLA2 (Figure 3D; Figure S6, Supporting Information). Reconstruction of the nucleus in VSMCs under 15% stretch and static conditions showed that cells subjected to 15% stretch had a larger surface area and greater spreading compared to those under static conditions (Figure S7, Supporting Information). Immunofluorescence scanning along the Z axis showed endogenous cPLA2 mainly in the nucleoplasm of VSMCs under static conditions; but in cells under 15% stretched, significant cPLA2 signals were detected at the nuclear membrane, colocalized with the nuclear envelope protein Lamin B1 (Figure 3E,F). This finding is consistent with hypotonic conditions. ArAc content, used to evaluate cPLA2 activation, showed that VSMCs transfected with WT cPLA2 and exposed to 15% stretch for 3 h had increased ArAc production; this effect was abolished by transfection with Y96A or V97A mutants (Figure 3G). Mechanical force rapidly induced ArAc production, with significant increases at 15 min (Figure 3H).

Nuclear lamins, fibrous proteins in type V intermediate filaments, maintain nuclear membrane tension.^[^ ^44^ ^]^ Interfering with Lamin A/C expression has been shown to facilitate cPLA2 activation induced by hypotonic stress.^[^ ^28^ ^]^ We assessed cPLA2 activation under static or stretch conditions in cells with LMNA knockdown (encoding Lamins A/C) or scrambled siRNA. Exposure to 15% stretch increased cPLA2 translocation to the nuclear membrane and ArAc production, indicating cPLA2 activation; these effects were further enhanced by LMNA knockdown, which could increase nuclear membrane tension (Figure 3I,J). As actin cytoskeleton force transmission is central to mechanosensing and mechanotranduction,^[^ ^44^ ^]^ we explored the role of actin polymerization in cPLA2 mechanosensing of cyclic stretch. Cells treated with SMIFH2 (a formin inhibitor preventing formin‐driven actin polymerization) showed attenuated stretch‐induced cPLA2 activation (Figure S8A,B, Supporting Information). Altogether, the findings suggest that high‐magnitude cyclic stretch actives cPLA2 by increasing nuclear membrane tension.

### 2.4 Nuclear Mechanosensor cPLA2 Mediates Downregulation of CPT1B Expression and FAO in Response to High‐Magnitude Cyclic Stretch

**Figure 4:**
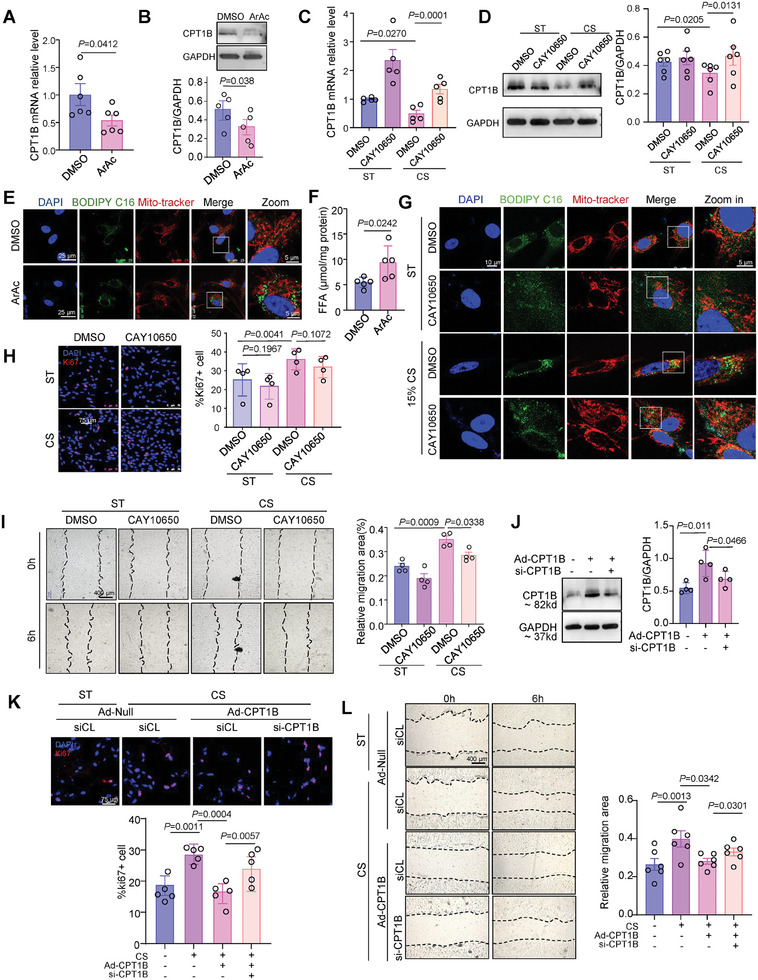
Nuclear mechanosensor cPLA2 mediates downregulation of CPT1B expression and FAO in response to high‐magnitude cyclic stretch. A) Quantitative RT‐qPCR assessment of CPT1B expression in VSMCs treated with ArAc (10 µmol L^−1^) for 12 h. *n* = 6 biological replicates. B) Western blotting analysis of CPT1B protein levels in VSMCs treated with ArAc for 24 h. *n* = 5 biological replicates. C) Quantitative RT‐qPCR assessment of CPT1B expression in VSMCs pretreated with cPLA2 inhibitor CAY10650(12 nmol L^−1^) for 3 h and subjected to 15% cyclic stretch (CS) or static (ST) for 12 h. *n* = 5 biological replicates. D) Western blotting analysis of CPT1B protein levels in VSMCs pretreated with CAY10650 for 3 h and subjected to 15% CS or ST for 24 h. *n* = 6 biological replicates. E) Representative BODIPY FL C16 fluorescence images of VSMCs treated with ArAc for 24 h. F) Free fatty acid (FFA) content in VSMCs treated with DMSO or ArAc for 24 h. *n* = 5 biological replicates. G, Immunofluorescent images of BODIPY FL C16 and mito‐tracker in VSMCs pretreated with CAY10650 and subjected to 15% CS or ST for 24 h. H,I) Cell proliferation (H) and migration (I) in VSMCs pretreated with CAY10650 and subjected to 15% CS or ST for 24 h. Migration areas and the Ki67‐positive cell ratio were analyzed using ImageJ software. *n* = 4 biological replicates. J) Western blotting and quantification showing the effectiveness of CPT1B overexpression and knockdown in VSMCs. *n* = 4 biological replicates. K,L) Cell proliferation (K) and migration (L) in VSMCs with CPT1B overexpression and knockdown subjected to 15% CS or ST for 24 h. *n* = 5 biological replicates (K), *n* = 6 biological replicates (L). Data were analyzed using unpaired Student *t*‐test (A, B, and F), paired Student *t*‐test (J) or Two‐Way ANOVA (C, D, H, I, and L). Error bars show ± SEM. Exact *p*‐values between the indicated groups are presented.

To assess the effects of cPLA2 activation on CPT1B expression, we measured the mRNA and protein levels of CPT1B and found that treatment with ArAc (HY‐109590, 10 µmol L^−1^) significantly decreased both (**Figure** **4**A,B). In VSMCs exposed to 15% stretch for 3 h or maintained under static conditions, treatment with CAY10650 (HY‐10801, 12 nmol L^−1^), a potent cPLA2 inhibitor, attenuated the downregulation of CPT1B mRNA and protein in response to the 15% stretch (Figure 4C,D). Fluorescence imaging with BODIPY FL C16 and FFA detection confirmed excessive accumulation of fatty acid in VSMCs treated with ArAc (Figure 4E,F). Furthermore, the accumulation of fatty acids outside of mitochondria in the form of lipid droplets under 15% stretch was reduced by CAY10650 treatment (Figure 4G).

Next, we examined cell behavior. Ki67 staining and scratch assays showed that 15% stretch promoted VSMC proliferation and migration, but this effect was attenuated by inhibiting cPLA2 with CAY10650 (Figure 4H,I). To clarify the mediatory role of CPT1B in cell proliferation and migration in response to cyclic stretch, we overexpressed CPT1B by infecting cells with Ad‐CPTB adenovirus. We then knocked down CPT1B by transfecting si‐CPT1B. Western blot analysis confirmed the effectiveness of both CPT1B overexpression and knockdown (Figure 4J). Scratch assays and Ki67 staining revealed that CPT1B overexpression attenuated stretch‐induced VSMC proliferation and migration, while subsequent CPT1B knockdown reactivated these processes (Figure 4K,L). Collectively, these findings support the conclusion that cPLA2 mediates the downregulation of CPT1B expression, impairment of FAO, and excessive VSMC proliferation and migration in response to high‐magnitude cyclic stretch.

### 2.5 cPLA2 Activation Downregulates CPT1B Expression by Acting on the Transcription Factor YY1

**Figure 5:**
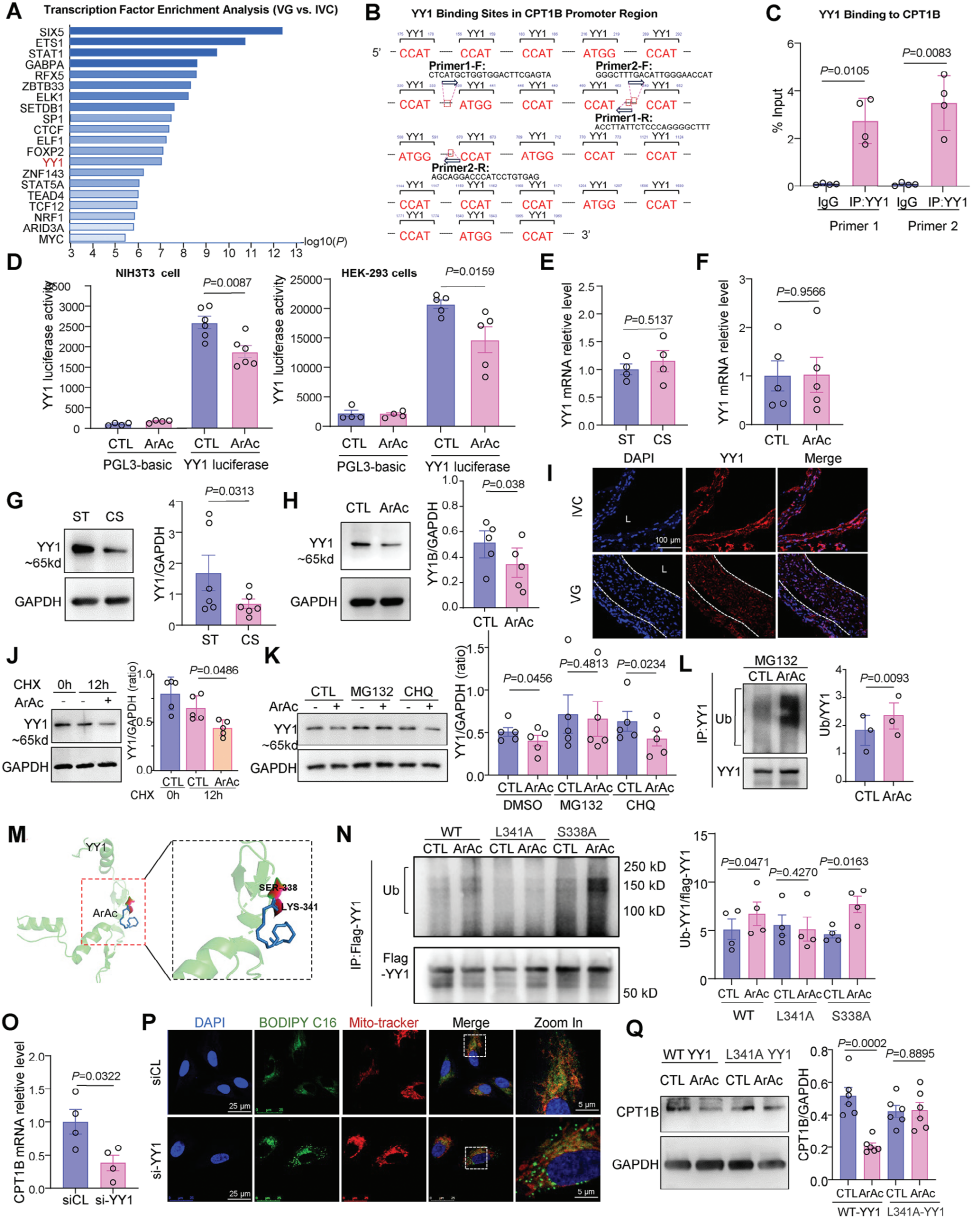
cPLA2 activation downregulates CPT1B expression by acting on the transcription factor YY1. A) Transcription factor enrichment analysis of differentially expressed genes in transcriptomics of VGs versus IVCs analyzed using the CHEA3 online tool. B) Analysis of YY1 transcription factor binding sequences within the putative human promoter regions (Chromosome 22, NC_000022.11) of CPT1B. YY1‐binding elements and the ChIP‐qPCR primers are indicated. C) ChIP assay to detect binding of YY1 to the CPT1B promoter regions in VSMCs. *n* = 4 biological replicates. D) Detection of YY1 transcriptional activity by luciferase assay in NIH3T3 and HEK‐293 cells treated with ArAc for 24 h. Luciferase activity was normalized to the β‐galactosidase activity. *n* = 5 biological replicates (NIH3T3 cells), *n* = 6 biological replicates (HEK‐293 cells). E,F) Quantitative RT‐qPCR showing YY1 mRNA expression in VSMCs under 15% CS (E) or ArAc treatment (F) for 12 h compared to control groups, respectively. *n* = 5 biological replicates. G,H) Western blotting analysis of YY1 protein expression in VSMCs under 15% CS (G) or ArAc treatment (H) for 24 h compared to control groups. I) Immunofluorescent images of DAPI (nucleus) and YY1 in the IVCs and 6‐week VGs. J) Stability of YY1 in VSMCs in the presence or absence of ArAc. VSMCs were treated with protein synthesis inhibitor cycloheximide (CHX, 10µmol L^−1^) for 12 h, and the YY1 levels relative to GAPDH were assayed by Western blotting. *n* = 5 biological replicates. K) VSMCs were treated with CHX (10 µmol L^−1^) or lysosomal pathway inhibitor chloroquine (CHQ, 100 µmol L^−1^) under ArAc treatment for 24 h. The YY1 levels relative to GAPDH were assayed by Western blotting. *n* = 5 biological replicates. L) Western blotting detection of the ubiquitination level of YY1 in VSMCs. Cells were treated with ArAc for 24 h, and MG132 (20 µmol L^−1^) was added to the media and incubated for 10 h. Immunoblotting with an anti‐ubiquitin and ubiquitin‐like proteins antibody (Ub) indicates the ubiquitination level of YY1. *n* = 3 biological replicates. M) Molecular docking prediction showing that ArAc may interacts directly with YY1 through key amino acid ser‐338 and lys‐341. N) Cells were transfected with YY1‐flag‐WT, YY1‐flag‐L341A, or YY1‐flag‐L341A mutation plasmids, and Western blotting detected the ubiquitination level of flag‐YY1 in cells. *n* = 4 biological replicates. O) Quantitative RT‐qPCR assessment of CPT1B expression in VSMCs transfected with si‐CL or si‐YY1 for 24 h. *n* = 6 biological replicates. *n* = 4 biological replicates. P) Immunofluorescent images of DAPI (nucleus), BODIPY FL C16 and Mito‐tracker in VSMCs transfected with si‐CL or si‐YY1. Q) Western blotting analysis of CPT1B protein levels in cells treated with ArAc for 24 h. NIH3T3 cells were transfected with YY1‐flag‐WT, YY1‐flag‐L341A, or YY1‐flag‐L341A plasmids. *n* = 6 biological replicates. Data were analyzed using unpaired Student *t*‐test (C, G, H, J and K), unpaired Mann‐Whitney test (D, E, F, and N), paired Mann‐Whitney test (L) or Two‐Way ANOVA (Q). Error bars show ± SEM. Exact *p*‐values between the indicated groups are presented.

We sought to determine how CPT1B expression is regulated by cPLA2. We performed transcription factor enrichment analysis of differentially regulated genes in VGs versus IVCs transcriptomics using the CHEA3 online tool and listed the top 20 candidates (**Figure** **5**A). Analysis of the putative promoter regions of CPT1B using the JASPAR online tool revealed 23 potential binding sites for YY1, which is the greatest one among the 20 candidates (Figure 5B; Figure S9, Supporting Information). Chromatin immunoprecipitation (ChIP) assay with anti‐YY1 antibody demonstrated direct binding of YY1 with the CPT1B promoter in VSMCs (Figure 5C). We hypothesized that YY1 might play an important role in the regulation of CPT1B by cyclic stretch. Supporting this hypothesis, the YY1 luciferase reporter assay demonstrated that the transcriptional activity of YY1 was inhibited by ArAc in NIH3T3 and HEK‐293 cells (Figure 5D). Upon further investigation, we found that YY1 mRNA expression was not significantly affected by cyclic stretch or ArAc treatment (Figure 5E,G). However, the protein level of YY1 was indeed downregulated by cyclic stretch or ArAc treatment (Figure 5G,H). Consistently, immunofluorescent staining confirmed the downregulation of YY1 protein levels in VGs relative to IVCs (Figure 5I).

To study how YY1 protein was downregulated by cyclic stretch or cPLA2 activation in terms of ArAc production, we treated cells with the protein synthesis inhibitor cycloheximide (CHX, 50 µg mL^−1^). In VSMCs treated with ArAc, YY1 protein expression was reduced compared to DMSO‐treated controls (Figure 5J), suggesting that ArAc inhibits YY1 protein expression by accelerating its degradation. To determine the mechanism of ArAc‐induced YY1 degradation, we treated VSMCs with inhibitors for either the lysosomal pathway (CHQ) or the proteasomal pathway (MG132), and then assessed YY1 degradation. Western blotting indicated that treatment with MG132 attenuated ArAc‐induced reduction in YY1 levels, whereas CHQ did not (Figure 5K). These results indicated that ArAc‐induced YY1 degradation in VSMCs might be mediated by the proteasomal pathway. Further supportive evidence came from results showing that 15% stretch increased YY1 ubiquitination (Figure 5L). To unveil the structural basis of the relationship between ArAc and YY1 ubiquitination, we conducted interaction predictions between YY1 and ArAc using molecular docking with the AutoDock Vina online program. Serine‐338 and lysine‐341 of YY1 were identified as key amino acid residues within their interacting interface (Figure 5M). We generated mutations of serine‐338 and lysine‐341 to alanine (L341A and S338A). Western blotting indicated that L341A attenuated ArAc‐induced YY1 ubiquitination (Figure 5N), confirming the importance of YY1‐ArAc interaction in regulating YY1 ubiquitination. We then assessed regulation of YY1 on CPT1B expression and lipid metabolism. The results showed that YY1 knockdown inhibited CPT1B mRNA expression and led to the accumulation of fatty acids outside mitochondria in the form of lipid droplets (Figure 5O,P). The influence of YY1 mutation, L341A, on CPT1B downregulation in response to ArAc was also confirmed by Western blotting assays (Figure 5Q). Taken together, these findings demonstrate that the mechanical activation of cPLA2 downregulates CPT1B expression by acting on the transcription factor YY1.

### 2.6 Inadequate FAO Increases Nuclear Membrane Tension, Orchestrating the Activation of cPLA2

**Figure 6:**
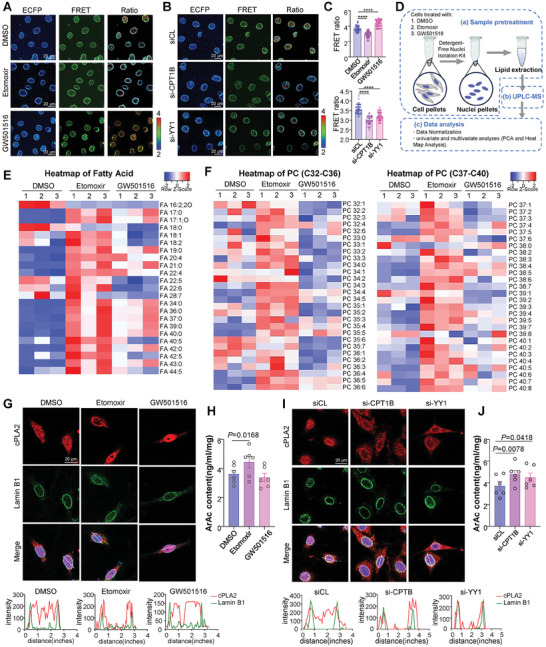
Inadequate FAO increases nuclear membrane tension, orchestrating the activation of cPLA2. A–C) FRET assay to measure the nuclear membrane tension in cells with the indicated treatments. Representatives images showing ECFP, FRET and FRET/ECFP ratio in VSMCs treated with etomoxir or GW501516 for 24 h (A). Representative images showing ECFP, FRET and FRET/ECFP ratio in VSMCs transfected with si‐CPT1B, si‐YY1 or si‐CL for 48 h (B). FRET ratio was calculated by the open‐source software Fluocell (C). Data were analyzed using parametric unpaired Mann‐Whitney test. D) Scheme of the nuclear purification and lipidomic experiments. E) Heatmaps showing the abundance of long chain FAs in cell nuclei under DMSO, etomoxir and GW501516 treatment. F) Heatmaps showing the abundance of PCs (PC32‐PC40) in cell nuclei under DMSO, etomoxir and GW501516 treatment. The heatmap scale ranges from −2 to 2, with red indicating higher FAs and PCs abundance. G) Representative immunofluorescent images of cPLA2 and Lamin B in VSMCs treated with etomoxir or GW501516 for 24 h. Quantified cPLA2 distribution by fluorescence intensity profiling. H, Detection of ArAc content in VSMCs treated with etomoxir or GW501516 for 24 h. *n* = 6 biological replicates. I) Representative immunofluorescent images of cPLA2 and Lamin B in VSMCs transfected with si‐CPT1B, si‐YY1 and si‐CL for 48 h. Quantified cPLA2 distribution by fluorescence intensity profiling. J) Detection of ArAc content in VSMCs transfected with si‐CPT1B, si‐YY1, and si‐CL for 48 h. *n* = 6 biological replicates. Data were analyzed using unpaired Student *t*‐test. Error bars show ± SEM. Exact *p*‐values between the indicated groups are presented.

Given that the nuclear membrane largely consists of a lipid bilayer, we investigated whether aberrant FAO feedback affects nuclear membrane tension. Using the LAPS biosensor, we found that knockdown of CPT1B or YY1, as well as FAO inhibition by etomoxir, increased nuclear membrane tension, as indicated by a decrease in the FRET ratio (**Figure** **6**A–C). Conversely, enhancing FAO with GW501516 decreased nuclear membrane tension (Figure 6A,C).

Unsaturated fatty acids (UFAs), which contain double bonds, are degraded through FAO into acetyl‐CoA,^[^ ^45^ ^]^ and are key components of phosphatidylcholine (PC) in nuclear membranes.^[^ ^46^ ^]^ PCs with unsaturated fatty acyl chain increase membrane fluidity and tension.^[^ ^47^ ^]^ To assess lipidomic changes of the nucleus during FAO dysfunction, we performed a UPLC‐MS‐ based lipidomic analysis on nuclei treated with etomoxir or GW501516 (Figure 6D). PCA and heatmap analysis revealed distinct lipidomic signatures for DMSO, etomoxir, and GW501516 (Figure S10A,B, Supporting Information). Etomoxir treatment led to an enrichment of long chain UFAs in nuclei, including linoleic acid (FA 18:2), oleic acid (FA 18:1) and ArAc (FA 20:4) (Figure 6E). Furthermore, PCs with longer and unsaturated fatty acyl chains (PC 32‐PC 40) were elevated in etomoxir‐treated nuclei (Figure 6F). In contrast, GW501516 treatment reduced the levels of some UFAs and unsaturated PCs, such as PC 32:1, PC 32:2, and PC 33:2 (Figure 6E,F). These findings suggest dynamic remodeling of membrane components, leading to increased nuclear membrane tension, during FAO inhibition. Immunofluorescence of cPLA2 and measurements of ArAc indicated that knockdown of CPT1B or YY1, or inhibition of FAO with etomoxir, induced translocation of cPLA2 to the nuclear membrane and increased ArAc production (Figure 6G–I), both suggesting cPLA2 activation. Enhancing FAO with GW501516 resulted in a suppression of cPLA2 activation, although this effect was not significant (Figure 6G,I). These data suggest that impaired FAO might further increase nuclear membrane tension, thereby orchestrating the activation of cPLA2.

### 2.7 Interfering with the cPLA2‐CPT1B Axis Alleviates VSMC Proliferation and Neointimal Formation in VGs

**Figure 7:**
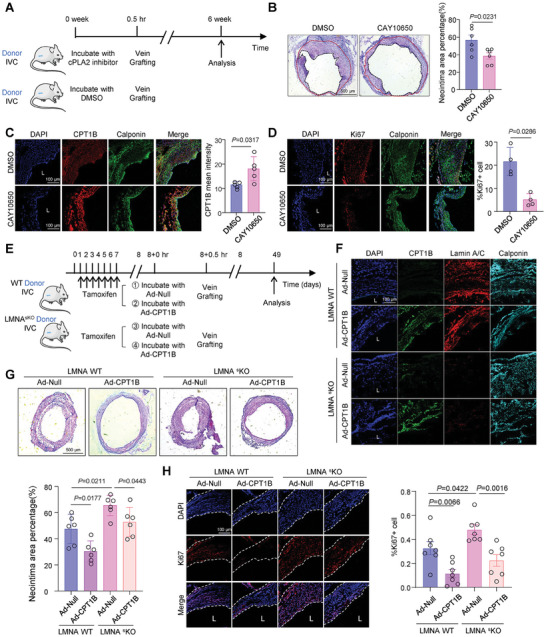
Interfering with the cPLA2‐CPT1B axis alleviates VSMC proliferation and neointimal formation in VGs. A) Strategy to inhibit cPLA2 activation in mice VGs and the experimental outline. B) Representative H&E staining of the cross sections of 6‐week VGs pretreated with DMSO or CAY10650 for 30 min at 37 °C before implantation. Quantitative analysis of neointimal area is shown. *n* = 6 mice. C) Representative immunofluorescent images of DAPI (nucleus), CPT1B and smooth muscle cell marker Calponin in 6‐week VGs pretreated with DMSO or CAY10650. Quantification of immunofluorescence intensity was conducted using ImageJ software. *n* = 5 mice. D) Representative immunofluorescent images of DAPI (nucleus), Ki67 and Calponin in 6‐week VGs treated with DMSO or CAY10650. Quantification of the Ki67‐positive cell ratio was conducted using ImageJ software. *n* = 4 mice. E) Strategy to overexpress CPT1B in VGs of LMNA wildtype (WT) and smooth muscle‐specific LMNA knockout (LMNA^sKO^) mice and the experimental outline. F) Immunofluorescence of CPT1B and Lamin A/C indicating the efficiency of CPT1B overexpression and Lamin A/C knockout. G) Representative H&E staining of the cross sections of VGs treated with DMSO or CAY10650 in (F). *n* = 6 mice. H) Representative immunofluorescent images of DAPI (nucleus) and Ki67 in 6‐week VGs in (F). Quantification of the Ki67‐positive cell ratio was conducted using ImageJ software. *n* = 7 mice. Data were analyzed using unpaired Student *t*‐test (B), unpaired Mann‐Whitney test (C and D) or Two‐Way ANOVA (G and H). Error bars show ± SEM. Exact *p*‐values between the indicated groups are presented.

To explore the in vivo relevance of our in vitro findings, we conducted vein grafting in mice, targeting cPLA2, nuclear membrane tension, and CPT1B. In some experiments, IVCs were collected from donor WT mice and soaked in saline supplemented with either the cPLA2 inhibitor CAY10650 (10 µmol L^−1^) or the control DMSO treatment for 30 min at 37 °C before implantation (**Figure** **7**A). Six weeks later, VG fragments were harvested from the recipient WT mice for morphological analysis, which indicated a reduced neointima area in VGs treated with the cPLA2 inhibitor compared to those treated with DMSO (Figure 7B). Immunofluorescence analysis revealed that incubation with CAY10650 increased CPT1B expression in Calponin‐labeled VSMCs and decreased the number of Ki67‐positive proliferative cells in VGs compared to DMSO incubation (Figure 7C,D).

In other experiments, to genetically create veins with increased nuclear membrane tension, we generated VSMC‐specific Lamin A/C knockout mice (LMNA^flox/flox^, Myh11‐CreERT2^+^, referred to LMNA sKO), along with littermate controls (LMNA WT, LMNA^flox/flox^, Myh11‐CreERT2^−^). Lamin A/C was specifically inactivated in VSMCs following tamoxifen injection for 7 days. Subsequently, vein grafting was performed on the 8^th^ day. To manipulate CPT1B expression in vivo, IVCs from the donors were soaked in saline supplemented with either Ad‐CPT1B adenovirus or control adenovirus Ad‐Null (2.50 × 10^10^ plaque‐forming units mL^−1^) for 30 min at 37 °C before implantation (Figure 7E). Immunofluorescence confirmed the effectiveness of CPT1B overexpression and Lamin A/C knockout (Figure 7F). Hematoxylin and eosin staining and Ki67 staining indicated that using LMNA sKO mice as donors aggravated neointimal hyperplasia and VSMC proliferation in VGs, whereas virus‐mediated CPT1B overexpression alleviated neointimal hyperplasia and VSMC proliferation (Figure 7G,H). These results suggest that targeting the cPLA2‐CPT1B pathway could be an effective intervention to prevent excessive VG remodeling.

## 3 Discussion

Currently, CABG surgery is the gold standard for the treating patients with complex multivessel coronary artery disease and/or left main disease.^[^ ^7^, ^48^ ^]^ However, VG restenosis and low long‐term graft patency remain significant limitations of CABG that have not been fully addressed. In this study, we identified a mechanism underlying VG restenosis (Figure S11, Supporting Information), demonstrating that the mechanosensory cPLA2 induces FAO deficiency in response to arterial mechanical stretch. Our evidence shows that high‐magnitude cyclic stretch activates cPLA2, leading to the generation of ArAc. ArAc promotes the ubiquitination and degradation of the transcription factor YY1, thereby inhibiting CPT1B expression. This inhibition of CPT1B expression and FAO levels promotes the proliferation and migration of VSMCs and neointimal hyperplasia. Our results suggest that targeting the cPLA2‐CPT1B pathway could be a potential intervention to prevent neointimal formation and improve VG patency.

Neointimal hyperplasia involves inflammation, cell proliferation, migration, phenotypic transition, and remodeling of the extracellular matrix in the vascular wall.^[^ ^49^ ^]^ Abnormal proliferation and migration of VSMCs are key cellular events in VG neointimal formation.^[^ ^11^, ^50^ ^]^ These processes are influenced by the complex arterial mechanical microenvironment, where VSMCs experience cyclic stretch due to arterial dilation during the cardiac cycle.^[^ ^51^ ^]^ While the cyclic strain in vein walls is relatively small (≈2–4%), it can reach up to 20% under pathological conditions such as vein grafting and hypertension.^[^ ^51^, ^52^ ^]^ Arterial cyclic stretch is a crucial inducer of behavioral changes in venous VSMCs, playing a vital role in excessive VG remodeling.^[^ ^35^, ^52^ ^]^

Our current study not only demonstrates the significant role of cyclic stretch in regulating VG VSMC behavior but also reveals how VSMCs perceive and transduce these mechanical forces. We focused on cPLA2, an enzyme in the phospholipase A2 family that hydrolyzes the sn‐2 acyl bond of glycerophospholipids. Under the action of cPLA2, ArAc is released from membrane phospholipids, promoting inflammation, cell signaling, and carcinogenesis.^[^ ^53^ ^]^ Previous studies have shown that cyclic stretch increases cPLA2 mRNA levels and induce rapid phosphorylation of cPLA2 in epithelial cells through the activation of specific upstream signaling pathways.^[^ ^54^, ^55^ ^]^ However, these studies did not prove that cPLA2 senses cyclic stretch. Recent research identified cPLA2 as a nuclear mechanosensory protein, capable of sensing increased nuclear membrane tension induced by hypotonic conditions and geometrical restriction.^[^ ^28^, ^29^, ^56^ ^]^ Consistent with prior studies, we demonstrate that cyclic stretch activates cPLA2 by increasing the nuclear membrane tension, causing it to insert into the nuclear envelope. Using the cPLA2 inhibitor CAY10650, we showed that cPLA2 inhibition attenuated stretch‐induced proliferation and migration of VSMCs, highlighting the critical role of cPLA2 in mediating the cellular behavioral changes in responses to mechanical forces. How are these mechanical forces transmitted to the nucleus? The nuclear envelope contains a transmembrane protein complex composed mainly of Nesprins and SUN proteins, which transmits extracellular stress to the nuclear lamina via the cytoskeleton‐LINC complex.^[^ ^25^ ^]^ Our findings, obtained by using the formin inhibitor SMIFH2 to disrupt force transmission by the actin cytoskeleton, provide supportive evidence for the transmission of force from extracellular environment to the nucleus.

Recent studies have highlighted the importance of cellular metabolism in cardiovascular homeostasis and diseases.^[^ ^11^, ^57^ ^]^ CPT1B, an isoform found mainly in muscle tissue, is the key rate‐limiting enzyme of FAO.^[^ ^33^ ^]^ FAO has been shown to be boosted by treatment with platelet‐derived growth factor‐BB human (PDGF‐BB) in pulmonary artery VSMCs, despite PDGF‐BB being known to induce VSMC proliferation and migration.^[^ ^58^ ^]^ Conversely, highly proliferative endothelial cells and endogenous neural stem cells exhibit low FAO levels compared to nonproliferative cells;^[^ ^59^, ^60^ ^]^ inhibition of FAO by CPT1B inactivation stimulates cardiomyocyte proliferation, enabling heart regeneration in adult mice.^[^ ^19^ ^]^ This seeming paradoxical behavior of FAO prompted us to speculate that impeded FAO might be related to VSMC proliferation and migration, as well as VG hyperplasia. This speculation is in line with evidence that mechanical stimulation through hypertensive pressure alone is sufficient to cause rapid lipid droplet accumulation and alterations in lipid metabolism and VSMC phenotype.^[^ ^20^, ^61^ ^]^ How does FAO dysregulation influence cell proliferation? FAO impairment in mitochondria causes excessive production of ROS due to lipid accumulation and decreased NADPH levels.^[^ ^38^, ^39^ ^]^ Increased ROS with consequent oxidative damage has been shown to promote vascular neointima formation by stimulating the proliferation of VSMCs.^[^ ^37^ ^]^ ROS may induce VSMC proliferation by activating signaling molecules in the mitogen‐activating protein kinases (MAPK) pathway, including extracellular regulated kinase1/2 (ERK1/2).^[^ ^62^, ^63^ ^]^ ROS also promotes the transcription of growth‐related genes through transcription factors such as AP‐1 and NFκB,^[^ ^64^ ^]^ and induces secretion of growth factors such as cyclophilin A,^[^ ^65^ ^]^ thus enhancing cell proliferation. Noted that we cannot rule out other factors besides ROS, such as YY1 that has been reported to inhibit VSMCs proliferation.^[^ ^66^ ^]^ Oscillations of the nuclear lipid pool are also strictly associated with cell cycle progression.^[^ ^67^ ^]^ Consistently, our findings demonstrated that FAO inhibition enhanced cell proliferation by reducing mito‐ROS clearance and increasing mito‐ROS generation. Moreover, by analyzing transcriptomics of VGs versus IVCs and cellular lipidomics in VSMCs under high‐ or low‐magnitude stretch, our current study revealed that high‐magnitude cyclic stretch causes FAO inhibition in VSMCs. Importantly, we provide evidence that cPLA2 mediates the cyclic stretch‐induced FAO inhibition, which subsequently promotes cell proliferation and migration. We demonstrated that treating venous segments with Ad‐CPT1B adenovirus or CAY10650 inhibited VG neointimal formation within six weeks of implantation, likely due to their inhibitory effects on excessive VSMC proliferation and migration. Our findings support our speculation and suggest that improving impaired FAO has a beneficial effect on preventing VG restenosis.

The nuclear membrane is predominantly composed of phospholipids, with PC accounting for over 50%. PC is made up of saturated and UFAs, phosphoric acid, and choline.^[^ ^68^ ^]^ The composition of phospholipid and the degree of unsaturation significantly influence membrane fluidity,^[^ ^47^, ^69^ ^]^ suggesting that fatty acid metabolism may affect membrane fluidity and thus membrane tension. Using the LAPS membrane tension biosensor and cPLA2 fluorescent imaging, we demonstrated that knockdown of key molecules involved in force‐regulated signaling, such as CPT1B or YY1, increases nuclear membrane tension, thereby promoting cPLA2 activation. Similarly, FAO inhibition increases nuclear membrane tension, which also triggers cPLA2 activation. To further investigate lipid changes in the nuclear membrane, we performed a lipidomic analysis of cell nuclei. This analysis revealed that etomoxir treatment led to an increase in long‐chain UFAs and PCs, suggesting enhanced nuclear membrane fluidity and tension. Our findings point to a positive feedback loop involving nuclear membrane tension and lipid metabolism, implying that this feedback mechanism plays a critical role in aggravating disease progression. While we focused on changes in FAs and PCs, the notable alterations in other lipid classes, such as sphingomyelins, phosphatidylethanolamines, phosphatidylinositols, and cholesteryl esters, warrant further investigation.

YY1 is a zinc finger transcription factor that can activate or repress gene expression depending on its cofactors.^[^ ^70^ ^]^ It has protective effects in blood vessels, with overexpression alleviating neointimal hyperplasia after balloon injury of rat carotid arteries.^[^ ^66^, ^71^ ^]^ Recent studies have shown that YY1 is deeply involved in dysregulated cellular metabolism,^[^ ^72^ ^]^ acting as an activator of genes linked to FAO through direct recruitment to their promoters.^[^ ^73^ ^]^ However, the potential regulation of YY1 on CPT1B and the effect of mechanical stretch on YY1 expression or function have not been documented so far. In our study, we found that cyclic stretch and ArAc reduce YY1 protein levels by influencing it ubiquitination. Ubiquitination‐mediated degradation of YY1 is important for its function.^[^ ^74^ ^]^ Our results showed that YY1 downregulation is mediated by its ubiquitination, which in turn inhibits CPT1B expression. Additionally, our data indicated that YY1 interacts with ArAc at the lysine‐341 site, and this interaction promotes YY1 ubiquitination. These findings expand the understanding of post‐transcriptional regulation of YY1 and provide new insights into its role in VSMC functional regulation. Overall, this study elucidates how mechanical stimulation affects VSMCs during VG hyperplasia and reveals the role of the cPLA2‐CPT1B axis in this process.

## 4 Experimental Section

### Cell Culture

Primary human umbilical artery smooth muscle cells (SMCs) were isolated from the umbilical cords of healthy newborns. The umbilical cords were obtained with patient consent and approved by the ethics committee (2023PHB170‐001). All experiments utilized SMCs at passages 3 to 8, cultured in F12 Ham Kaighn's Modification (N6760‐10 × 1L, Sigma Aldrich) supplemented with 20% fetal bovine serum (FBS, 900‐008, Gemini) and 10% SMC growth medium (311‐500; Cell Applications) at 37 °C with 5% CO_2_. For stretch experiments, SMCs were incubated in medium supplemented with 10% FBS. To increase CPT1B expression, cells were infected with adenovirus harboring the human CPT1B gene (Ad‐CPT1B) or a null‐adenovirus (Ad‐Null) at a concentration of 3.0 × 10^6^ plaque‐forming units (PFU) mL^−1^. To inhibit CPT1B or YY1 expression, cells were transfected with siRNA specifically targeting human CPT1B or YY1 mRNA (si‐CPT1B or si‐YY1, 20 nmol L^−1^) or a scrambled siRNA (si‐CL) for 48 h. To inhibit cPLA2 activation, cells were pretreated with CAY10650 (HY‐10801, MedChemExpress) at 12 nmol L^−1^ in culture medium for 1 h before mechanical exposure. To simulate cPLA2 activation, cells were pretreated with arachidonic acid at 10 µmol L^−1^ in culture medium for 12 h. To stimulate cellular FAO, cells were incubated with the PPARδ agonist GW 501 516 (GC15318‐5, GlpBio) at 5 µmol L^−1^ in culture medium for 12 h. To inhibit CPT1B activation, cells were incubated with etomoxir (HY‐50202, MedChemExpress) at 5 µmol L^−1^ in culture medium for 12 h. To disrupt the actin cytoskeleton, cells were incubated with the formin inhibitor SMIFH2 (T23372, TargetMol) at 20 µmol L^−1^ in culture medium for 12 h. To inhibit intracellular ROS production, cells were incubated with N‐Acetylcysteine (HY‐B0215, MedChemExpress) at 5 mmol L^−1^ in culture medium for 24 h.

### Cell Cyclic Stretch Experiment

The cyclic stretch loading model of cultured SMCs was established using the TMS Boxer Cyclic Stretch Culture System (ATMS‐Boxer, Taiwan). SMCs were seeded on type‐I collagen‐coated (Biocoat#354 236) flexible silicone base films (ATMS#CH2020). The cells on the flexible silicone base films were then incubated overnight at 37 °C in a humidified atmosphere with 5% CO_2_. When cell confluence reached 70%–80%, SMCs were subjected to uniaxial cyclic stretch (5% or 15% cyclic strain) for 1, 3, 6, 12, or 24 h at a frequency of 1 Hz. Cells cultured under static conditions served as the control group.

### Animals

All animal studies were conducted in accordance with the guiding principles of the Animal Care and Use Committee of Peking University and approved by the ethics committee of the Medical Department of Peking University (LA2022144). C57BL/6J wild‐type (WT) mice were obtained from the animal center of Peking University. To generate mice with conditional knockdown of LMNA specific to SMCs, the CreERT2‐loxP‐mediated recombination system was employed. Mice carrying the LMNA coding region flanked by loxP sites (LMNA ^flox/flox^) were obtained from Jackson Laboratories (stock number 02 6284).^[^ ^75^ ^]^ Mice with the CreERT2 transgene under the control of the SMC‐specific myosin heavy chain promoter (Myh11‐Cre) were also obtained from Jackson Laboratories (stock number 01 9079). In the Myh11‐CreERT2 mice, the bacterial artificial chromosome (BAC) transgene was inserted into the Y chromosome, so only male mice expressed Cre recombinase. To eliminate the influence of sex and age in this study, WT male C57BL/6J mice (8–12 weeks, 20–30 g) were used during the experiments.

### Statistical Analysis

Statistical analyses were performed using GraphPad Prism version 8.0.1. Data were presented as means ± SEM from at least three independent experiments. Normality was tested using the Shapiro‐Wilks test, and equal variances were assessed using the Brown‐Forsyth test. For comparison between two groups, the Student *t*‐test was used if variances were equal; otherwise, nonparametric tests (Mann‐Whitney test) were used. For comparisons among more than two groups, one‐way or two‐way ANOVA followed by Tukey's post hoc test was used, or the Kruskal‐Wallis test and Dunn's multiple comparison test for nonparametric data. Exact *p*‐values are provided in the figures, with *p* < 0.05 considered statistically significant.

The data and methods supporting this study are available from the corresponding author upon reasonable request. An extended methods section is provided in the Supporting Information.

## Conflict of Interest

The authors declare no conflict of interest.

## Author Contributions

J.Z. designed and supervised the project. L.F. initiated the project, designed and executed the experiments, and analyzed the data. Y.T., J.L., Y.L., Y.X., J.L., H.L., W.Y., Y.G., and W.P. assisted with the experiments. J.Z. and L.F. wrote the manuscript. Q.P. supervised the FERT experiments and performed the analysis. T.Z. provided the human specimens and contributed valuable discussions on the clinical relevance. All authors reviewed and revised the manuscript.

## Supporting information



Supporting Information

## Data Availability

The data that support the findings of this study are available in the supplementary material of this article.
