# Supplementary material for: Mechanical Activation of cPLA2 Impedes Fatty Acid β‐Oxidation in Vein Grafts
Source: Adv Sci (Weinh). 2024 Nov 26;12(3):2411559. doi: 10.1002/advs.202411559 (PMC11744522; doi:10.1002/advs.202411559)

**Supporting Information**

**Mechanical Activation of cPLA2 Impedes Fatty Acid β-Oxidation in Vein Grafts**

Linwei Fan, Yuanjun Tang, Jian Liu, Yueqi Liu, Yiwei Xu, Jiayu Liu, Han Liu, Wei Pang, Yuxuan Guo, Weijuan Yao, Tao Zhang*, Qin Peng*, Jing Zhou*

**Expanded Methods**

**Animals**

All animal studies were conducted in accordance with the guiding principles of the Animal Care and Use Committee of Peking University and approved by the ethics committee of the Medical Department of Peking University (LA2022144). C57BL/6J wild-type (WT) mice were obtained from the animal center of Peking University. To generate mice with conditional knockdown of LMNA specific to SMCs, we employed the CreERT2-loxP-mediated recombination system. Mice carrying the LMNA coding region flanked by loxP sites (LMNA ^flox/flox^) were obtained from Jackson Laboratories (stock number 026284)^[61]^. Mice with the CreERT2 transgene under the control of the SMC-specific myosin heavy chain promoter (Myh11-Cre) were also obtained from Jackson Laboratories (stock number 019079). In the Myh11-CreERT2 mice, the bacterial artificial chromosome (BAC) transgene was inserted into the Y chromosome, so only male mice expressed Cre recombinase. To eliminate the influence of sex and age in this study, we used WT male C57BL/6J mice (8-12 weeks, 20-30g) during the experiments. Primers for genotyping are listed in Table S2.

**Vein Grafting**

Mice were anesthetized by intraperitoneal injection of 1.25% avertin, and vein grafting was performed using the previously described "cuff" technique. Briefly, both ends of the right common carotid artery were ligated with 8-0 silk thread. The blood vessel was then cut between the two ligation points. A cuff (Portex, London, UK) was affixed to the artery with a hemostatic clip and then secured with an 8-0 suture. The inferior vena cava (IVC) was collected from donor mice, incubated with Ad-Null/Ad-CPT1B (2.51×10^10^ pfu/mL) and DMSO/CAY10650 (10 μmol/L), or left untreated for 30 minutes, and then transplanted onto the right common carotid artery using the cuff technique. The vein was fixed on the cuff with 8-0 silk thread. Upon releasing the arterial clamp, significant blood flow was observed, indicating successful vascular pulsation in the vein graft (VG). Six weeks post-operation, the mice were perfused with PBS and 4% paraformaldehyde. VG and IVC samples were harvested. Mice with thrombosis in the vein grafts were excluded from the study. The blood vessels were embedded in OCT embedding medium (SAKURA) and stored at -40°C.

**Viruses, siRNAs, Plasmids, and Transfection**

Adenoviruses expressing the human CPT1B gene (Ad-CPT1B) and a control adenovirus (Ad-Null) were purchased from HANBIO, China. For amplification, adenoviruses were transfected into HEK-293A cells. Two days post-transfection, viral supernatants were harvested and filtered using a 0.45 µm polyethersulfone filter.

siRNAs for gene knockdown and scrambled siRNA were purchased from Tsingke. The sequences of siRNAs targeting CPT1B, YY1, and Lamin A/C are listed in Table S3. The pCMV3-untagged-human CPT1B plasmid was purchased from Sino Biological (HG19086-UT). WT and R605A Lamin A phosphorylation sensor (LAPS) plasmids were constructed as described previously^[36]^. The pcDNA3.1-3xFlag-human YY1 plasmid was purchased from You Biological (F110813). This plasmid was used to generate L341A and S338A point mutants using the Fast Mutagenesis System (FM111-02; TRANS). The full-length human cPLA2 ORF was cloned into SgfI and MluI sites of the pEnter/Flag&His vector (WZ Biosciences) to create the pEnter/Flag&His-cPLA2 plasmid. For generating the cPLA2-EYFP plasmid, the cPLA2 fragment was subcloned into SacI-BamHI sites of the pEYFP-N1 vector. The cPLA2-EYFP plasmid was used to generate V97A and Y96A point mutants using the Fast Mutagenesis System (FM111-02; TRANS). The YY1 luciferase reporter plasmid and the cPLA2-mCherry plasmid were constructed using the ClonExpress Ultra One Step Cloning Kit (C115-01; Vazyme) to ligate sequences into the 8×GTIIC luciferase reporter and pEnter/Flag&His-cPLA2 plasmids opened by restriction digestion. Plasmid DNA was amplified in Escherichia coli and purified using the TIANprep Mini Plasmid Kit (Tiangen). All primers and sequences used for plasmid construction are listed in Table S4. All generated plasmid sequences were confirmed by DNA sequencing.

When cells reached 70% to 80% confluence, the culture medium was replaced with OPTI-MEM medium (Thermo Fisher). Plasmids/siRNAs were mixed with Lipofectamine 2000 (Invitrogen) according to the manufacturer’s instructions, incubated for 20 minutes, and then added directly to the cell culture medium. The effects of transfection were assessed 24-48 hours post-transfection.

**Lipidomics Analysis**

Cell pellets from cells subjected to 5% and 15% cyclic stretch for 24 hours were collected, ensuring similar cell numbers in each group through cell counting. Lipid metabolites were extracted following protein precipitation with methanol. A large-scale untargeted metabolomic analysis was conducted using a the untargeted ultra-performance liquid chromatography coupled to mass spectrometry (UPLC-MS). Cells were analyzed for a total of 6,013 lipid species, including fatty acyls, glycerolipids, glycerophospholipids, sphingolipids, sterol lipids, prenyl alcohol lipids, glycolipids, and polyketides.

Cell pellets from cells treated with DMSO, etomoxir or GW501516 for 24 hours were collected. The cell nuclei were then biochemically fractionated using a detergent-free nuclei isolation kit (NI-024, Invent Biotechnologies), and the nuclei lipids were extracted with isopropyl alcohol and subjected to the UPLC-MS analysis.

Raw data from LC-QTOF MS were processed using Skyline software for peak extraction. Differential analysis and visualization of the lipidomics data were performed using the MetaboAnalyst online platform.

**Radioisotope Detection of FAO Levels**

To measure cellular FAO levels, we utilized the principle that mitochondrial FAO converts [9,10-^3^H]-palmitic acid into ^3^H_2_O. This conversion allows the detection of ^3^H_2_O as a measure of FAO activity. The reaction mixture consisted of F12 Nutrient Mixture with 74 KBq/mL [9,10-^3^H]-palmitic acid (1.96 TBq/mol; NET043001MC; PerkinElmer, Waltham, MA) and 4% bovine serum albumin (without palmitic acid, Applygen). The treated SMCs were resuspended into a twelve-well plate and incubated with the reaction mixture at 30°C for 3 hours. Following the incubation, 2mol/L KCl-HCl solution was added to halt the reaction. The mixture was then washed with CHCl_3_ methanol (2:1) to extract the aqueous phase and remove lipids. The radioactivity of ^3^H in the aqueous phase was measured using a liquid scintillation counter (Tri-Carb 2500; Perkin-Elmer).

**Transcriptome Analysis**

Transcriptome data from VG and AVF were obtained from GEO datasets. Clean reads were aligned to the human reference genome (GRCh38.p13) in directed mode using HISAT2 software (http://ccb.jhu.edu/software/hisat2/index.shtml). Gene expression levels were calculated using the transcripts per million reads (TPM) method to identify differentially expressed genes (DEGs) between the two groups. Gene abundances were quantified using RSEM (http://deweylab.biostat.wisc.edu/rsem/), and DEGs were analyzed with DESeq2 (http://www.bioconductor.org/packages/release/bioc/html/DESeq2.html), identifying genes with *P*-values <0.05 and fold changes >2 or <0.5 as differentially expressed. Gene ontology (GO) and Kyoto Encyclopedia of Genes and Genomes (KEGG) enrichment analyses were conducted using the DAVID functional annotation tool.

**RNA Isolation and Quantitative RT-PCR**

Total RNA was extracted from cultured SMCs using TRIzol reagent (15596026; Thermo Fisher) following the manufacturer's instructions. The isolated RNA was reverse-transcribed into complementary DNA using Hifair III 1st Strand cDNA Synthesis SuperMix (11141ES60, Yeasen). Real-time PCR was performed with the 2×RealStar Power SYBR Mixture (A311-10, Genestar) using specific primer pairs. Gene expression analysis was conducted using the ΔΔCt method, with normalization against GAPDH. All primers used for RT-PCR are listed in Tables S3.

**Chromatin immunoprecipitation (ChIP)**

Cells were washed and crosslinked with 1% formaldehyde for 5 minutes, washed with ice‐cold PBS, and scraped into 0.5% SDS Lysis Buffer (with 1 mM DTT and protease inhibitors). The pellet was lysed and sonicated, 100 µL of mixture were used as an Input. The lysate was diluted, incubated with antibody against YY1 or IgG for overnight at 4 °C and then with 30 µl of protein A/G sepharose beads for another 3 h. The beads were washed, treated with RNase A (50 µg mL^−1^) and proteinase K (7.5 µL of 20 mg mL^−1^). Crosslinks were reversed at 65 °C overnight. DNA was extracted with FastPure Gel DNA Extraction Mini Kit (Vazyme, DC301-01) and subjected to PCR amplification. To verify the ChIP conditions, qPCR was performed on enriched DNA using primers against CPT1B promoter. The enrichment of immunoprecipitated DNA with anti‐YY1 antibody and negative control (IgG) versus input were calculated separately with the following formula: %Input=2 ^ (Ct Input-Ct ChIP)×IDF×100% .

**Luciferase Reporter Assay**

The YY1 luciferase reporter plasmid was constructed using the 8×GTIIC luciferase reporter, replacing the YAP response element with the YY1 response element (as detailed in Table S5). To assess YY1 activity, SMCs or NIH 3T3 cells were co-transfected with the YY1 luciferase reporter plasmid (or PGL3-basic) and the pSV-β-galactosidase plasmid using Lipofectamine 2000 (ThermoFisher) following the manufacturer’s instructions. Twenty-four hours post-transfection, the cells were treated with either arachidonic acid or DMSO for an additional 24 hours. Luciferase activity was then measured using the luciferase assay system (RG005, Beyotime) and normalized to β-galactosidase activity, which was assessed using o-nitrophenyl-β-D-galactopyranoside (0789, Amresco). The measurements were taken with a SpectroMax M3 Multimode microplate reader (Molecular Devices).

**Antibodies**

The following primary antibodies were used: Anti-Ki67 antibody (Ab15580, Abcam), CPT1B-specific polyclonal antibody (22170-1-AP, Proteintech), Lamin A/C polyclonal antibody (10298-1-AP, Proteintech), DYKDDDDK tag polyclonal antibody (20543-1-AP, Proteintech), Ubiquitin antibody (3933S, CST), YY1 rabbit polyclonal antibody (22156-1-AP, Proteintech), Anti-CPT1B antibody (ab134988, Abcam), Anti-cPLA2 antibody (bs-20212R, Bioss), and GAPDH (EASYBIO #BE0024).

**Western Blotting**

Cells were lysed in RIPA lysis buffer (MP015; Macgene) supplemented with phosphatase inhibitors (Apply #P12601). Protein concentration was determined using the Bicinchoninic Acid (BCA) assay with the Pierce™ BCA Protein Assay Kit (23250; Thermo Fisher). Equal amounts of protein were separated by SDS-PAGE, transferred to nitrocellulose membranes, and blocked with 5% skim milk or BSA in TBST for 1 hour. Membranes were then incubated with primary antibodies overnight at 4°C. The following day, membranes were incubated with secondary antibodies against rabbit/mouse IgG. Bands were visualized using the Molecular Imager ChemiDoc XRS+ System (Bio-Rad) or the Odyssey infrared imaging system (LI-COR Biosciences).

**Detection of Long-Chain Free Fatty Acids (FFA) in Veins**

FFA detection was performed using the Free Fatty Acid Assay Kit (S0215S, Beyotime) according to the manufacturer's instructions. VGs and IVCs were perfused with PBS, collected, and homogenized in BeyoLysis™ Buffer A for Metabolic Assay using an Ultra-Turrax homogenizer on ice. Homogenates were centrifuged at 12,000 × g for 10 minutes at 4°C, and the supernatant was collected for FFA detection. Absorbance was measured at 570 nm, and FFA content was calculated based on a standard curve. FFA levels were normalized to tissue total protein concentration and expressed as μmol/L/mg protein.

**YY1 Ubiquitination Assay**

Cells were lysed using a lysis buffer composed of 6 mol/L guanidine-HCl (pH 8.0), 0.1 mol/L Na_2_HPO_4_/NaH_2_PO_4_, and 10 mmol/L imidazole containing 5 mmol/L N-ethylmaleimide to prevent de-ubiquitination, with 1× protease inhibitor cocktail added. The lysates were incubated on ice for 30 minutes, followed by centrifugation at 12,000 rpm for 10 minutes at 4°C. The supernatant was incubated overnight at 4°C with the indicated antibodies or IgG on a rotating mixer. The immune complexes were then incubated with 20 μl of protein A/G agarose beads for 3 hours and washed three times with a wash buffer (1 mmol/L EDTA, 10 mmol/L Tris-HCl [pH 8.0], 150 mmol/L NaCl, 1% TritonX-100, 100 U/ml PMSF, and protease inhibitor cocktail). Each sample was boiled with equal volumes of 2×SDS loading buffer and then subjected to western blot analysis. To avoid interference from heavy chains, a goat anti-rabbit IgG (light chain) secondary antibody was used.

**Measurement for Cell Migration**

The migration of SMCs was assessed using a wound healing assay. Cells were seeded on silicon slides and subjected to mechanical stretching, followed by wounding along the stretching direction with a 200 μL pipette tip. Cells were pretreated with mitomycin-C (1 μg/mL), a DNA synthesis inhibitor, for 1 hour to inhibit cell proliferation and then cultured in low-serum (2% FBS) F12 medium. Migration areas were monitored at 6 hours post-wounding using microscopy. ImageJ software was used to measure the migration areas. Representative images were selected to accurately reflect the group average.

**Prediction of the Interaction Interface Between Protein and Compound**

The crystal structure of the YY1 protein (PDB ID: 1UBD) was obtained from the PDB database. The nucleic acid structure was removed, and the protein structure was modified to Autodock4 atom types. The structure was converted to pdbqt format using AutodockTools4.2.6. The 3D structure of the small molecule compound arachidonic acid was obtained from the PubChem database, protonated at pH 7.4, and converted to pdbqt format using OpenBabel3.1.1. Docking was performed using Autodock Vina 1.2.3 (https://autodock-vina.readthedocs.io/en/latest/introduction.html) with the following parameters: algorithm exhaustiveness of 16, nine candidate docking results, and an energy threshold of ±3 kcal/mol. Semi-flexible and blind docking methods were used. The optimal Vina binding affinity score for the 1UBD and ligand interaction was 4.628 kcal/mol. PyMOL was used to visualize the docking conformation, highlighting SER338C as a key residue forming a hydrogen bond with the ligand, and LYS341C as a key residue involved in π-alkyl and alkyl-alkyl interactions with the ligand.

**Arachidonic Acid (ArAc) Detection by Enzyme-Linked Immunosorbent Assay (ELISA)**

Samples for ELISA detection were prepared according to the manufacturer's instructions (Elabscience Biotechnology). Briefly, cells were gently washed with cold PBS, trypsinized, and centrifuged at 1000 × g for 5 minutes to collect the cells. The collected cells were washed three times with cold PBS. For resuspension, 150 µL of PBS (with protease inhibitors) was added per 10^6^ cells. Cells were lysed by repeated freezing in liquid nitrogen and thawing at 37°C. The lysate was centrifuged at 12,000 × g for 10 minutes at 4°C, and the supernatant was collected for detection using the ELISA kit. Absorbance was measured at 450 nm, and the arachidonic acid content was calculated based on the standard curve. Arachidonic acid contents were normalized to total protein concentration and expressed as ng/ml/mg protein.

**Immunofluorescence, Live Cell Imaging, and Quantification**

For fixed tissues, OCT-embedded tissues were quickly frozen and sliced. SMCs seeded on silicone base films were transferred to slides through adhesion. For immunofluorescence, cells and tissues were washed with PBS, fixed in 4% paraformaldehyde for 15 minutes, permeabilized with 0.25% Triton X-100 in PBS for 10 minutes, and incubated with blocking buffer (3% BSA in PBS) for 1 hour at room temperature. Primary antibodies were diluted 1:200 in PBS, and cells or tissues were incubated overnight at 4°C. After washing, samples were incubated with secondary antibodies (Alexa Fluor 488 or Alexa Fluor 555 conjugated goat anti-rabbit/-mouse IgG) for 1 hour at room temperature. Nuclei were stained with DAPI. For live cell imaging, cells transfected with fluorescent protein plasmids were placed in glass-bottom dishes and stained with Hoechst 33342 for 10 minutes. Fluorescence images of both fixed sections and live cells were visualized using a confocal microscope (Leica TCS-SP8 and TCS-SP8 STED 3X). Images were analyzed using ImageJ, with nuclear surface area calculated via 3D reconstruction using Leica Application Suite X 3D Analysis software. Subcellular fluorescence intensity was measured, and the Pearson colocalization coefficient was calculated using ImageJ.

**Assessment of Intracellular ROS Production**

Cells were incubated with culture medium containing 2'-7'-Dichlorodihydrofluorescein diacetate (DCFH-DA, 17.5 µmol/L) and MitoSOX Red (5 µmol/L) for 30 minutes and then washed with PBS and fixed with 4% PFA. Using a 40X objective, each coverslip was exposed at 485-nm excitation for 10 seconds and the emitted light was passed through a 530-nm barrier filter. DCFH-DA mean fluorescence intensity were quantified using ImageJ software.

**BODIPY FL C16**

BODIPY FL C16 (4,4-difluoro-5,7-dimethyl--bora-3a,4a-diaza-s-indacene-3-hexadecanoic acid) is a fluorescent palmitate analogue. SMCs were incubated with medium containing 1 μmol/L BODIPY FL C16 for 2 hours. After washing with medium without BODIPY FL C16, cells were incubated for 1 hour to allow for fatty acid metabolism. Mitochondria were labeled with 50 nM MitoTracker Red CMXRos for 30 minutes before imaging. For tissues, 100 µL of 200 µmol/L BODIPY FL C16 was injected via the tail vein. Six hours post-injection, VGs and autologous control IVCs were harvested for frozen sectioning. Distribution and accumulation of BODIPY FL C16 were visualized by fluorescence imaging and quantified using ImageJ software.

**FRET Imaging**

FRET imaging was captured using a Dragonfly Confocal Microscopy System with a seven-laser and EMCCD camera. ECFP and FRET channels were excited by a 445 nm laser, and emissions were collected using 478 nm and 571 nm filters, respectively. The FRET ratio was calculated using the open-source software Fluocell (http://github.com/lu6007/fluocell).

**Statistical Analysis**

Statistical analyses were performed using GraphPad Prism version 8.0.1. Data are presented as means ± SEM from at least three independent experiments. Normality was tested using the Shapiro-Wilks test, and equal variances were assessed using the Brown-Forsyth test. For comparison between two groups, the Student t-test was used if variances were equal; otherwise, nonparametric tests (Mann-Whitney test) were used. For comparisons among more than two groups, one-way or two-way ANOVA followed by Tukey's post hoc test was used, or the Kruskal-Wallis test and Dunn's multiple comparison test for nonparametric data. Exact *P*-values are provided in the figures, with *P* < 0.05 considered statistically significant.

**Supplement Tables**

| **Table S1. Original data of fatty acid content.** | | | | | | | | |
| --- | --- | --- | --- | --- | --- | --- | --- | --- |
|  | **15% CS** | | | | **5% CS** | | | |
|  | 1 | 2 | 3 | 4 | 1 | 2 | 3 | 4 |
| FA 16:0;O | 7918422 | 8547128 | 17209284 | 9314855 | 12653423 | 15057001 | 12604598 | 9503800 |
| FA 17:1 | 79834 | 48031 | 671018 | 90743 | 165427 | 108579 | 80926 | 67633 |
| FA 18:1 | 9479938 | 8771082 | 42462584 | 10920105 | 14940172 | 16093066 | 9178245 | 8211229 |
| FA 18:2 | 6456414 | 5465826 | 7154438 | 7833764 | 7037182 | 4755462 | 3099555 | 5177866 |
| FA 18:3 | 384500 | 319472 | 916583 | 629070 | 739626 | 431462 | 266868 | 448178 |
| FA 19:2 | 1189373 | 86377 | 921494 | 159221 | 312580 | 468923 | 327018 | 344255 |
| FA 20:0 | 1476447 | 2278481 | 1623794 | 2275289 | 2664145 | 2063861 | 1411732 | 1899761 |
| FA 20:1 | 627907 | 441343 | 3244312 | 878661 | 1439506 | 950472 | 551525 | 439763 |
| FA 20:2 | 408954 | 153886 | 3086119 | 478662 | 892002 | 1036379 | 951852 | 400497 |
| FA 20:3 | 956859 | 999153 | 33882520 | 753831 | 3062761 | 2523558 | 2460591 | 906039 |
| FA 20:4 | 10644015 | 9681047 | 2.24E+08 | 10252253 | 19497352 | 10670503 | 12944881 | 7947296 |
| FA 20:5 | 733886 | 615205 | 7993782 | 443702 | 1541948 | 819652 | 1241452 | 467421 |
| FA 21:2 | 3592792 | 1086561 | 833709 | 1882123 | 1035269 | 2692150 | 2067532 | 1960140 |
| FA 22:0 | 613246 | 891707 | 852075 | 592145 | 1076064 | 771006 | 490107 | 682439 |
| FA 22:1 | 209207 | 78887 | 670438 | 488597 | 433552 | 303741 | 320382 | 73946 |
| FA 22:4 | 3981625 | 4420269 | 25972956 | 4228802 | 10033670 | 6172819 | 6887950 | 3674475 |
| FA 22:5 | 3883647 | 2985897 | 27075538 | 2137268 | 7629292 | 4376450 | 7029240 | 2713271 |
| FA 22:6 | 13864563 | 8830201 | 1.07E+08 | 9596011 | 29560250 | 33571384 | 34535680 | 11879320 |
| FA 24:0 | 838204 | 1336111 | 1442916 | 1025938 | 1470228 | 649173 | 963001 | 1080622 |
| FA 24:1 | 135178 | 110800 | 518228 | 341822 | 489963 | 335057 | 263389 | 190053 |
| FA 24:3 | 40404 | 17677 | 735232 | 44645 | 208041 | 466437 | 344471 | 68653 |
| FA 24:4 | 53238 | 270830 | 2290753 | 1572162 | 300939 | 9229688 | 895608 | 218301 |
| FA 24:5 | 872842 | 558346 | 4475766 | 856436 | 2379754 | 2067856 | 2653645 | 872099 |
| FA 24:6 | 377208 | 283475 | 2336687 | 429624 | 1501360 | 1800192 | 2060076 | 396370 |
| FA 26:0 | 1609121 | 2470947 | 9800787 | 1315278 | 2118822 | 624849 | 786268 | 1717806 |
| FA 26:5 | 2116632 | 2913376 | 6774196 | 4603949 | 9584965 | 11541498 | 10321236 | 2776558 |
| FA 27:0 | 155772 | 325407 | 5629540 | 178570 | 162938 | 75649 | 231923 | 172705 |
| FA 28:0 | 1465954 | 1169430 | 6631071 | 674867 | 916143 | 391101 | 608836 | 742054 |
| FA 28:5 | 544719 | 236628 | 253909 | 378377 | 337165 | 264331 | 311956 | 243404 |
| FA 28:6 | 3240928 | 0 | 2735 | 497 | 39019 | 0 | 2693 | 637 |
| FA 30:0 | 111287 | 74332 | 676122 | 197728 | 183762 | 72984 | 127939 | 33136 |
| FA 30:5 | 90412 | 257746 | 526538 | 304810 | 708531 | 954977 | 711555 | 227987 |
| FA 30:6 | 794088 | 427656 | 416084 | 262210 | 477346 | 128574 | 307293 | 291867 |
| FA 30:7 | 1072497 | 384770 | 238840 | 133431 | 284828 | 138017 | 204037 | 108977 |
| FA 30:8 | 342680 | 158191 | 374326 | 347282 | 297534 | 669858 | 370267 | 297073 |
| FA 31:0 | 154889 | 80818 | 1203934 | 308717 | 252476 | 111062 | 182484 | 127047 |
| FA 32:8 | 361597 | 1575836 | 1069348 | 1988260 | 1057032 | 1114653 | 849823 | 774952 |
| FA 32:9 | 4071579 | 9174268 | 2621434 | 7347048 | 6771564 | 4934376 | 1541754 | 6131067 |
| FA 33:0 | 69749 | 29932 | 433892 | 37331 | 88369 | 398387 | 27220 | 0 |
| FA 34:0 | 24359 | 0 | 400038 | 50371 | 108733 | 549397 | 75717 | 0 |
| FA 36:10 | 2065715 | 1769281 | 812458 | 2558692 | 2845900 | 1456140 | 195660 | 2547206 |
| FA 36:5 | 1196244 | 1340175 | 1242652 | 910739 | 1145626 | 752087 | 248704 | 738833 |
| FA 38:10 | 66201 | 82573 | 165940 | 368950 | 32483 | 172343 | 41124 | 175103 |
| FA 38:5 | 12546305 | 10216627 | 15270927 | 8907563 | 10632633 | 7598870 | 6669557 | 7573298 |
| FA 38:6 | 15286 | 1003182 | 0 | 816432 | 410333 | 572483 | 86687 | 510449 |
| FA 38:8 | 421932 | 681158 | 9628144 | 523987 | 1252388 | 770041 | 444301 | 481083 |
| FA 40:0 | 101616 | 58910 | 192828 | 94214 | 141305 | 753364 | 275212 | 109558 |
| FA 40:5 | 5.86E+08 | 3.72E+08 | 7.11E+08 | 2.87E+08 | 4.92E+08 | 3.46E+08 | 3.28E+08 | 2.94E+08 |
| FA 42:0 | 119881 | 157663 | 218675 | 159782 | 126776 | 625727 | 343930 | 126417 |
| FA 42:5 | 9.41E+08 | 5.49E+08 | 9.54E+08 | 6.52E+08 | 8.71E+08 | 6.3E+08 | 6.45E+08 | 5.6E+08 |
| FA 42:6 | 1146354 | 4419147 | 774133 | 2805179 | 2087817 | 2198192 | 640532 | 2571726 |
| FA 42:7 | 540034 | 603170 | 2620985 | 590524 | 550314 | 438722 | 295103 | 613608 |
| FA 42:8 | 7324709 | 5761656 | 55422184 | 8544022 | 13214289 | 5280687 | 5273098 | 7547743 |
| FA 42:9 | 163151 | 131451 | 8845165 | 207375 | 700706 | 275471 | 131160 | 177706 |
| FA 44:10 | 168172 | 167956 | 66976 | 5882024 | 42311 | 0 | 0 | 816652 |
| FA 44:4 | 31745418 | 22554378 | 76340176 | 29558196 | 36792588 | 21797958 | 21849732 | 24682682 |
| FA 44:5 | 3E+08 | 2.08E+08 | 3.62E+08 | 2.66E+08 | 3.12E+08 | 2.04E+08 | 1.98E+08 | 2.07E+08 |
| FA 44:6 | 1493122 | 2096819 | 2199904 | 1902046 | 1798544 | 1059509 | 995735 | 1784735 |
| FA 44:7 | 231184 | 14993896 | 323799 | 10570459 | 5609299 | 6706442 | 1142060 | 7387120 |
| FA 44:8 | 0 | 2811309 | 0 | 1374833 | 435031 | 910768 | 0 | 855671 |

| **Table S2. The sequence of primers for mice genotyping.** | |
| --- | --- |
| Primer name | Sequence |
| Lamin A/C flox-F | AACCCAGCCTCAGAAACTGGTGGATG |
| Lamin A/C flox-R | GACAGCTCTCCTCTGAAGTGCTTGGA |
| SMMHC-Cre-F | TGACCCCATCTCTTCACTCC |
| SMMHC-Cre-R | AGTCCCTCACATCCTCAGGTT |

| **Table S3. The sequence of siRNAs.** | |
| --- | --- |
| Gene name | Sequence |
| CPT1B | GGAAGAGUACAUCUACCUUTT |
|  | AAGGUAGAUGUACUCUUCCTT |
| YY1 | GAUGAGAAUCUGAUCCUCGTT |
|  | CGAGGAUCAGAUUCUCAUCTT |
| Lamin A/C | AGCUGAAAGCGCGCAAUACCAAGAA |
|  | UUCUUGGUAUUGCGCGCUUUCAGCU |

| **Table S4. The sequence of primers used in PCR analysis.** | | | |
| --- | --- | --- | --- |
| NO. | Primer name | Gene name | Sequence |
| 1 | h-GAPDH-F | GAPDH | AAGGTGAAGGTCGGAGTCAA |
|  | h-GAPDH-R |  | AATGAAGGGGTCATTGATGG |
| 2 | h-CPT1B-F | CPT1B | TACAACAGGTGGTTTGACA |
|  | h-CPT1B-R |  | CAGAGGTGCCCAATGATG |
| 3 | h-MLYCD-F | MLYCD | AGCAACATCCAGGCAATCGT |
|  | h-MLYCD-R |  | GTTAATGGGGCCACCTGTGA |
| 4 | h-ACAT1-F | ACAT1 | GGAGGCTGGTGCAGGAAATA |
|  | h-ACAT1-R |  | TGCCTTTTCAATGGCTCCCT |
| 5 | h-ACSL1-F | ACSL1 | CCATGAGCTGTTCCGGTATTT |
|  | h-ACSL1-R |  | CCGAAGCCCATAAGCGTGTT |
| 6 | h-ACADL-F | ACADL | AGGGGATCTGTACTCCGCAG |
|  | h-ACADL-R |  | CTCTGTCATTGCTATTGCACCA |
| 7 | h-CPT2-F | CPT2 | CATACAAGCTACATTTCGGGACC |
|  | h-CPT2-R |  | AGCCCGGAGTGTCTTCAGAA |
| 8 | h-HADH-F | HADH | ACCAGGCAGTTCATGCGTT |
|  | h-HADH-R |  | ACGTGCTTGACGATTATCTTCTT |
| 9 | h-ECHS1-F | ECHS1 | TGAGCTTGCCATGATGTGTGA |
|  | h-ECHS1-R |  | AACAGGACAAATCTTGCTGACA |
| 10 | h-ACACa-F | ACACa | ATGTCTGGCTTGCACCTAGTA |
|  | h-ACACa-R |  | CCCCAAAGCGAGTAACAAATTCT |
| 11 | h-YY1-F | YY1 | GAGAGAACTCACCTCCTGAT |
|  | h-YY1-R |  | GGCTTCTCTCCAGTATGAAC |
| 12 | h-Lamin A/C-F | Lamin A/C | AGCAGCGTGAGTTTGAGAGC |
|  | h-Lamin A/C-R |  | AGACTGCCTGGCATTGTCC |
| 13 | h-CD36-F | CD36 | AAAGTCACTGCGACATGATTAATGG |
|  | h-CD36-R |  | AACGTCGGATTCAAATACAGCATAG |
| 14 | m- GAPDH -F | GAPDH | AGGTCGGTGTGAACGGATTTG |
|  | m- GAPDH -R |  | TGTAGACCATGTAGTTGAGGTCA |
| 15 | m-CPT1B-F | CPT1B | GAGACAGGACACTGTGTGGG |
|  | m-CPT1B-R |  | GTAGAGCTCCACGTCATCGG |
| 16 | m-ACAT1-F | ACAT1 | CAGGAAGTAAGATGCCTGGAAC |
|  | m-ACAT1-R |  | TGCAGCAGTACCAAGTTTAGTG |
| 17 | m-MLYCD-F | MLYCD | GCACGTCCGGGAAATGAAC |
|  | m-MLYCD-R |  | GCCTCACACTCGCTGATCTT |
| 18 | m-ACSL1-F | ACSL1 | TGCCAGAGCTGATTGACATTC |
|  | m-ACSL1-R |  | GGCATACCAGAAGGTGGTGAG |
| 19 | m-ACADL-F | ACADL | TTTCCTCGGAGCATGACATTTT |
|  | m-ACADL-R |  | GCCAGCTTTTTCCCAGACCT |
| 20 | m-CPT2-F | CPT2 | CAGCACAGCATCGTACCCA |
|  | m-CPT2-R |  | TCCCAATGCCGTTCTCAAAAT |
| 21 | m-HADH-F | HADH | TCAAGCATGTGACCGTCATCG |
|  | m-HADH-R |  | TCTCCAGCCTTAGGGTTTTCT |
| 22 | m-ECHS1-F | ECHS1 | TGTCCTGTTGAGACACTGGTG |
|  | m-ECHS1-R |  | ACAAACGCGGTCATCCCTTC |
| 23 | m-ACACa-F | ACACa | CTCCCGATTCATAATTGGGTCTG |
|  | m-ACACa-R |  | TCGACCTTGTTTTACTAGGTGC |
| 24 | ChIP1-F | CPT1B promoter | CTCATGCTGGTGGACTTCGAGTA |
|  | ChIP1-R |  | ACCTTATTCTCCCAGGGGCTTT |
| 25 | ChIP2-F | CPT1B promoter | GGGCTTTGACATTGGGAACCAT |
|  | ChIP2-R |  | AGCAGGACCCATCCTGTGAG |

| **Table S5. The sequence of primers for plasmid construction.** | | |  |
| --- | --- | --- | --- |
| Plasmids | Primer name | Sequence | |
| cPLA2‐EYPF | cPLA2-F | GGACTCAGATCTCGAGCTCAAATGTCATTTATAGATCCTTA | |
|  | cPLA2-R | CATGGTGGCGACCGGTGGATCCCGTGCTTTGGGTTTACTTAGAA | |
|  | YFP-F | GTAAACCCAAAGCAGGAGGAGGATCCATGGTGAGCAAGGGCGAGGA | |
|  | YFP-R | TGCTGGATATCTGCAGAATTCTTACTTGTACAGCTCGTCCATGCC | |
| cPLA2‐mCherry | cPLA2-F | CTTGGTACCGAGCTCGGATCCATGTCATTTATAGATCCTTAC | |
|  | cPLA2-R | CTCCTCCATTGCTTTGGGTTTACTTAGAAACTCC | |
|  | mCherry-F | AACCCAAAGCAATGGAGGAGGACAACATGGCC | |
|  | mCherry-R | TGCTGGATATCTGCAGAATTCTCCGGATCCACCACCGGT | |
| cPLA2-EYFP Point Mutants | cPLA2-F | CTTGGTACCGAGCTCGGATCCATGTCATTTATAGATCCTTACCAGCAC | |
|  | cPLA2-R | CTTGCTCACCATGGATCCTCCTCCTGCTTTGGGTTTACTTAGAA | |
|  | cPLA2-V97A-F | GTCCCTAGAGTTTCATCCATAGCATAATTG | |
|  | cPLA2-V97A-R | ATGGATGCCAATTATGCTATGGATGAAACT | |
|  | cPLA2-Y96A-F | GTTTCATCCATGACAGCATTGGCAT | |
|  | cPLA2-Y96A-R | AATGGATGCCAATGCTGTCATGGAT | |
| Flag-YY1  Point Mutants | YY1-F | CGCTCCCCGGCCATCTTGGCGGGTGGTCGCTCCCCGGCCATCTTGGCGGCTGGTGCCATCTT | |
|  | YY1-R | GATCAAGATGGCACCAGCCGCCAAGATGGCCGGGGAGCGACCACCCGCCAAGATGGCCGGGGAGCGGTAC | |
|  | L341A-F | AGCTTTTGTTGAGGCTTCAAAACTAAAACGA | |
|  | L341A-R | TCGTTTTAGTTTTGAAGCCTCAACAAAAGCT | |
|  | S338A-F | TGAGAGTTCAAAAGCAAAACGACACCAACT | |
|  | S338A-R | AGTTGGTGTCGTTTTGCTTTTGAACTCTCA | |


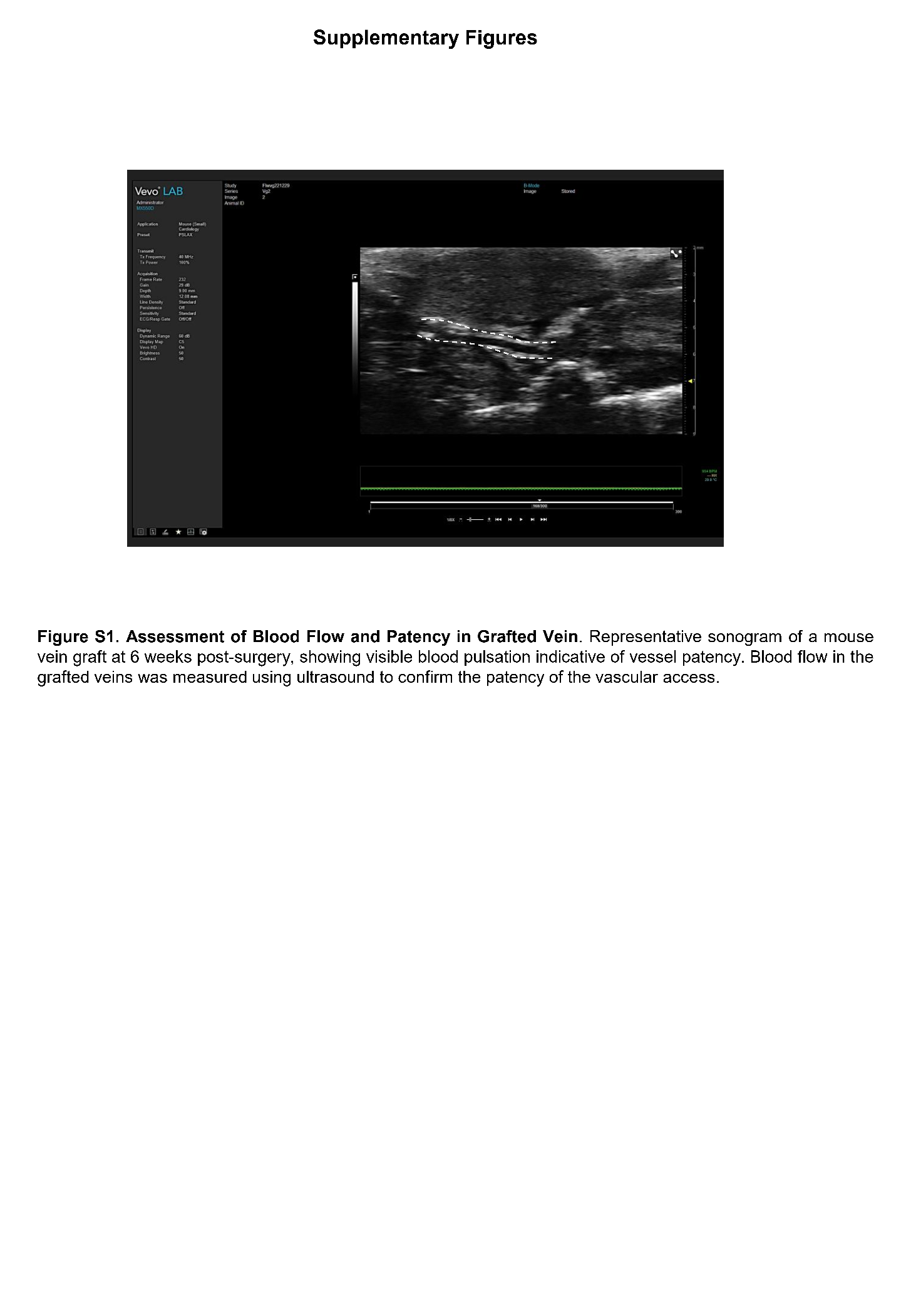


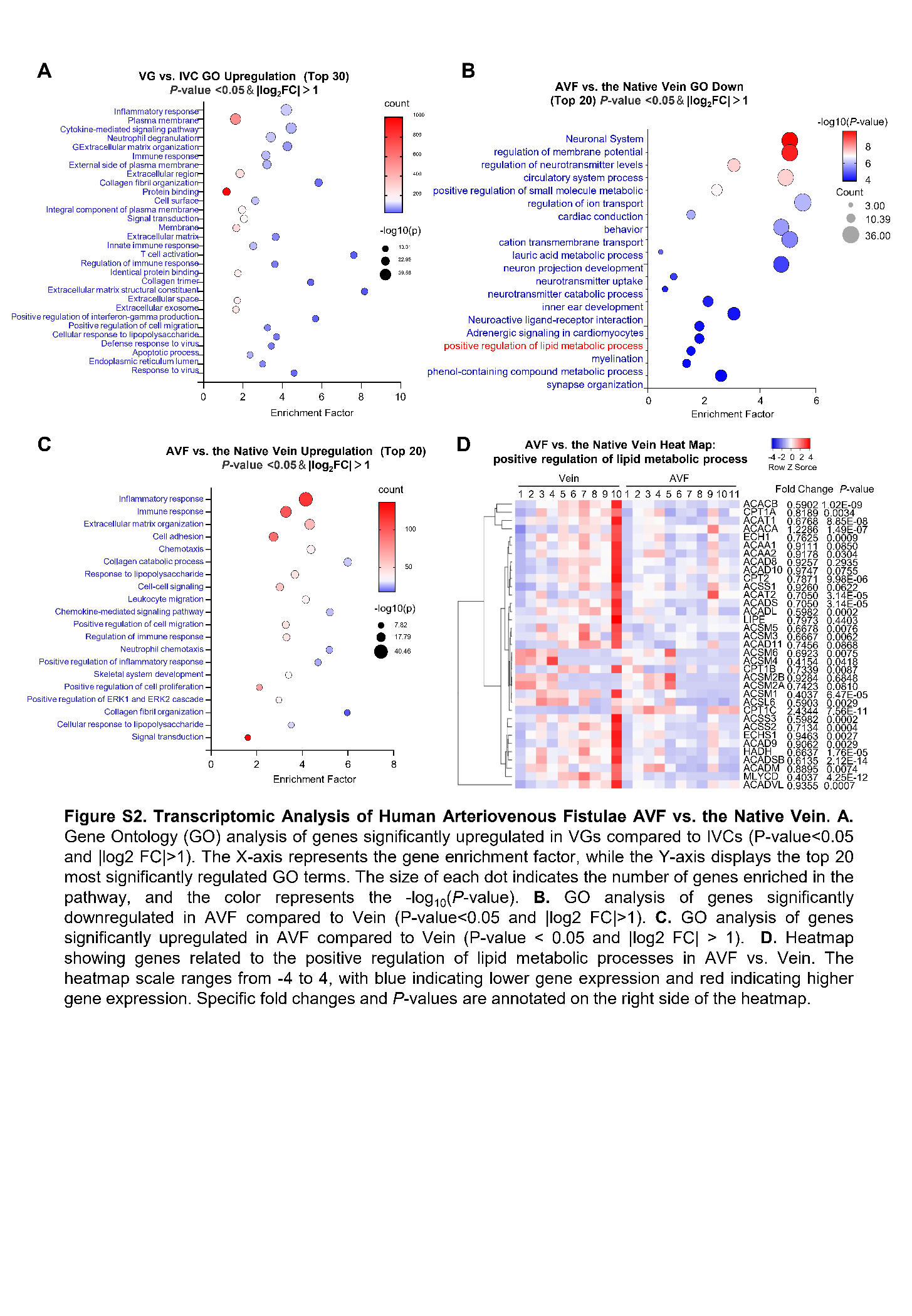


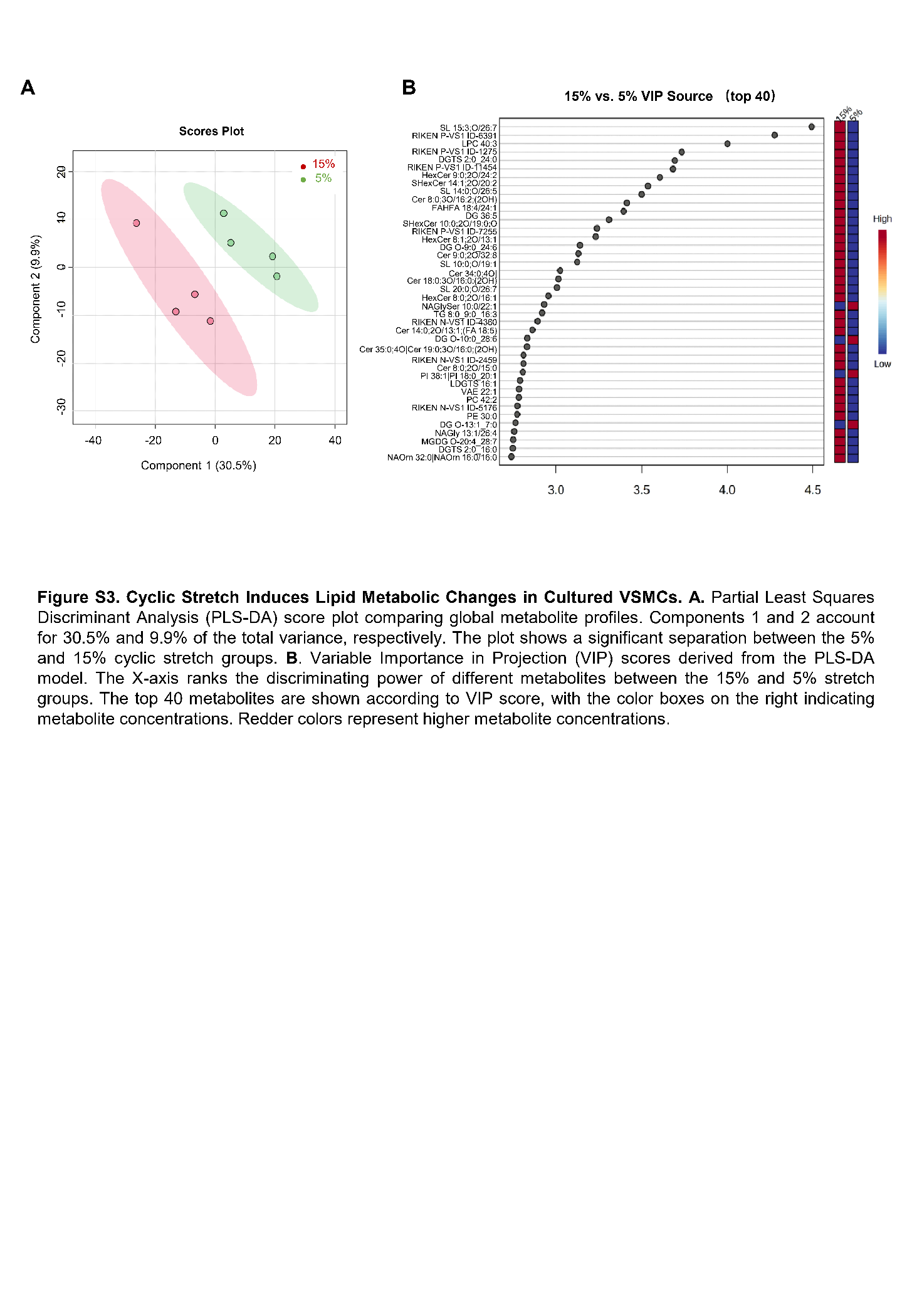


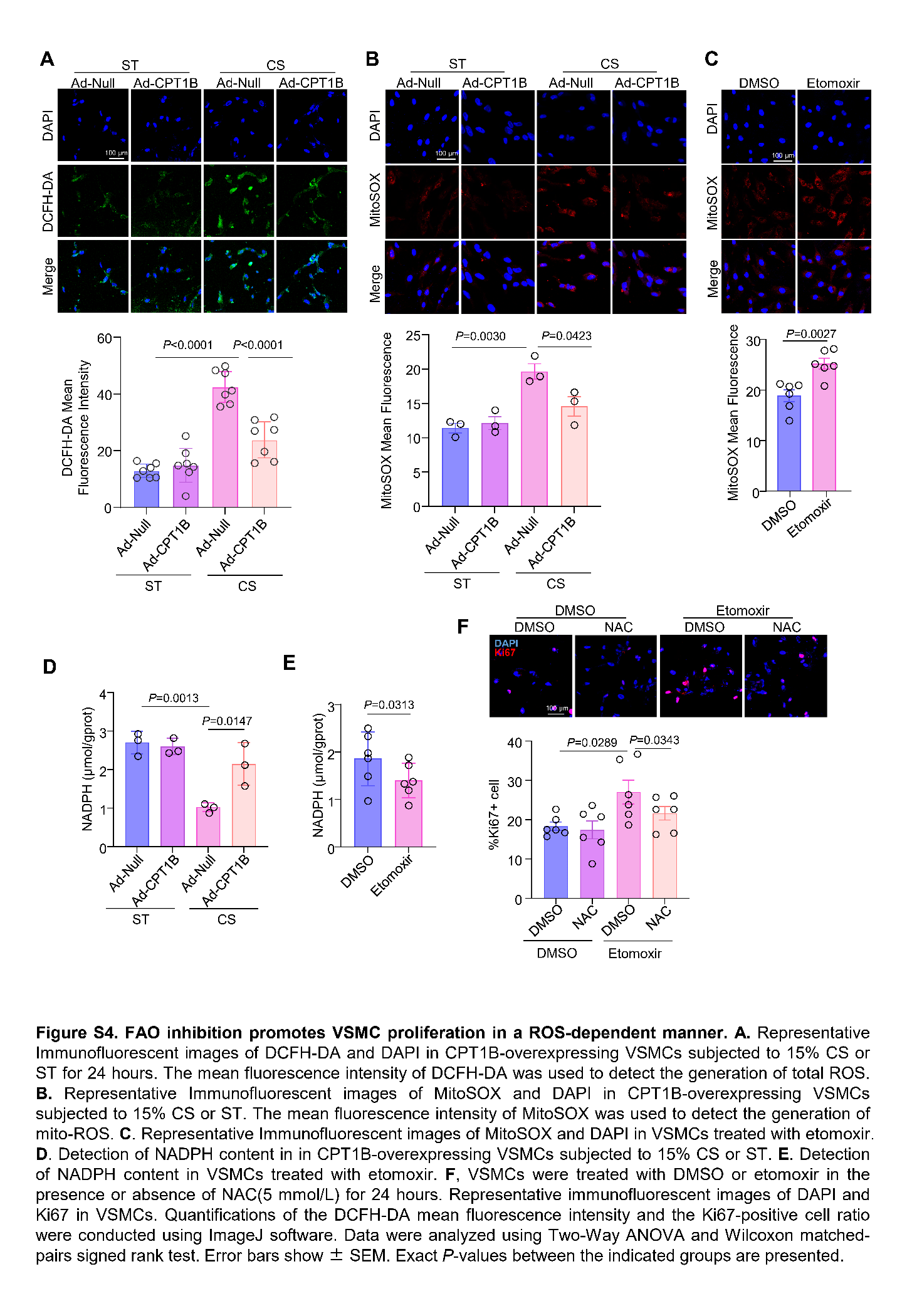


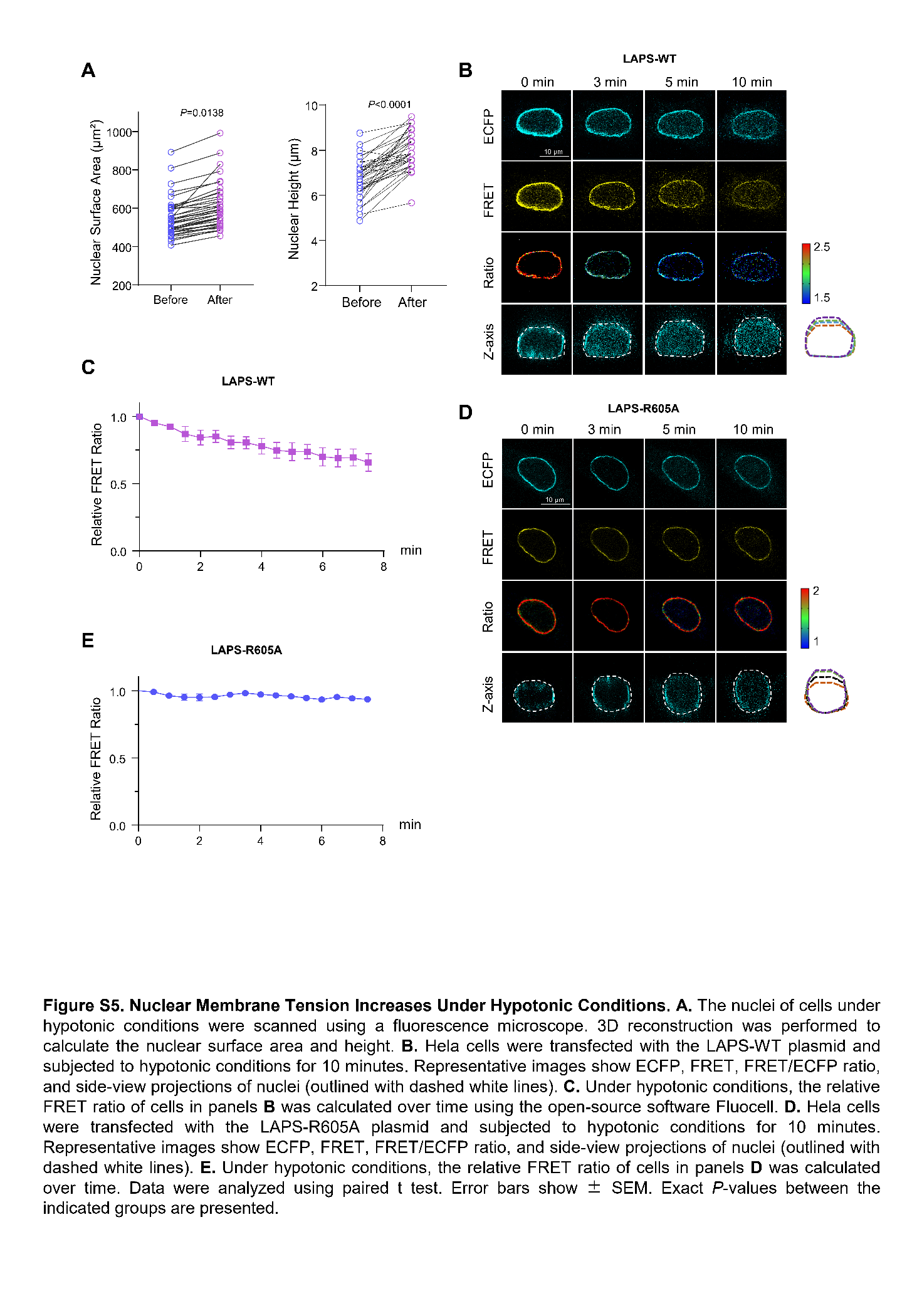


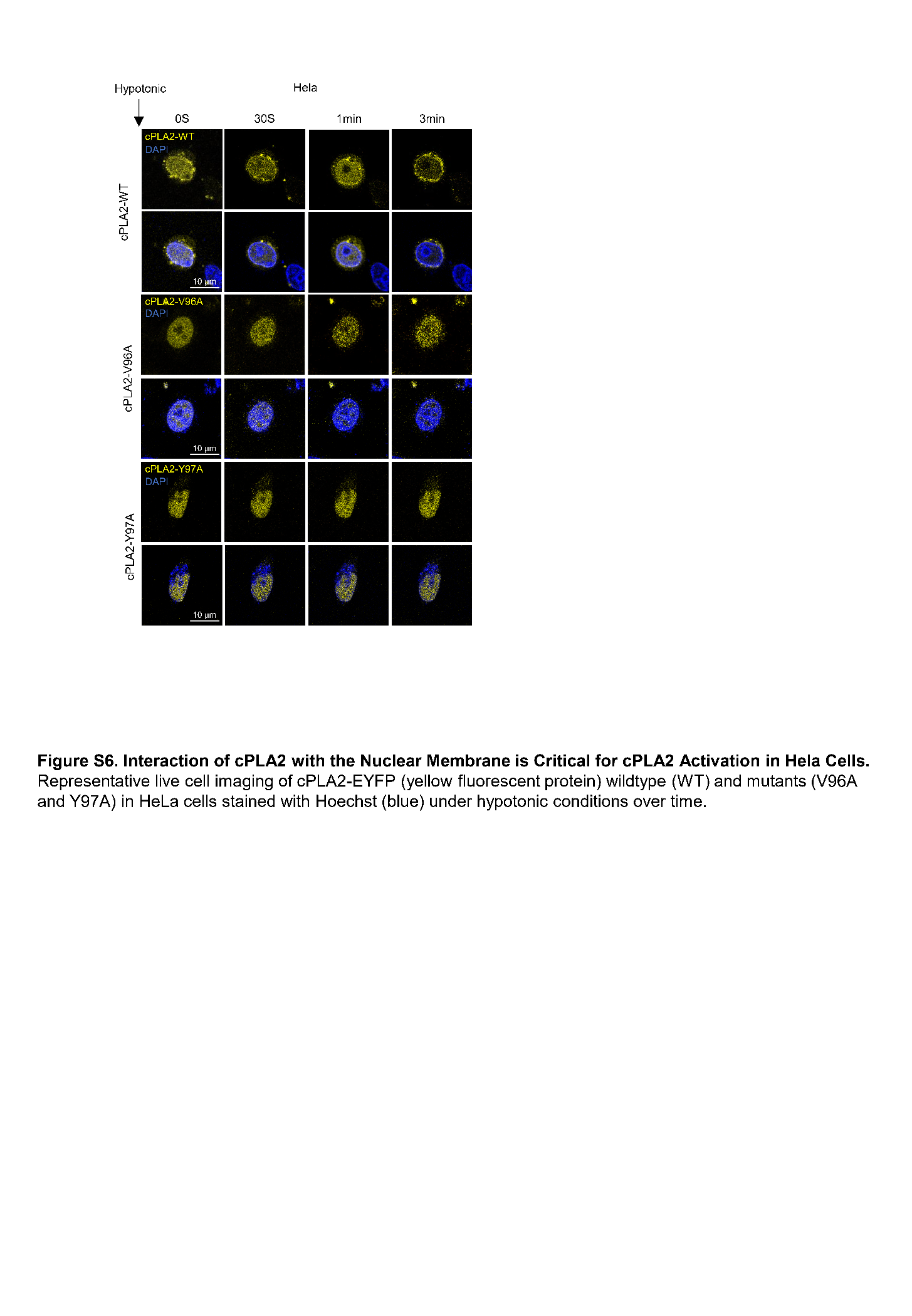


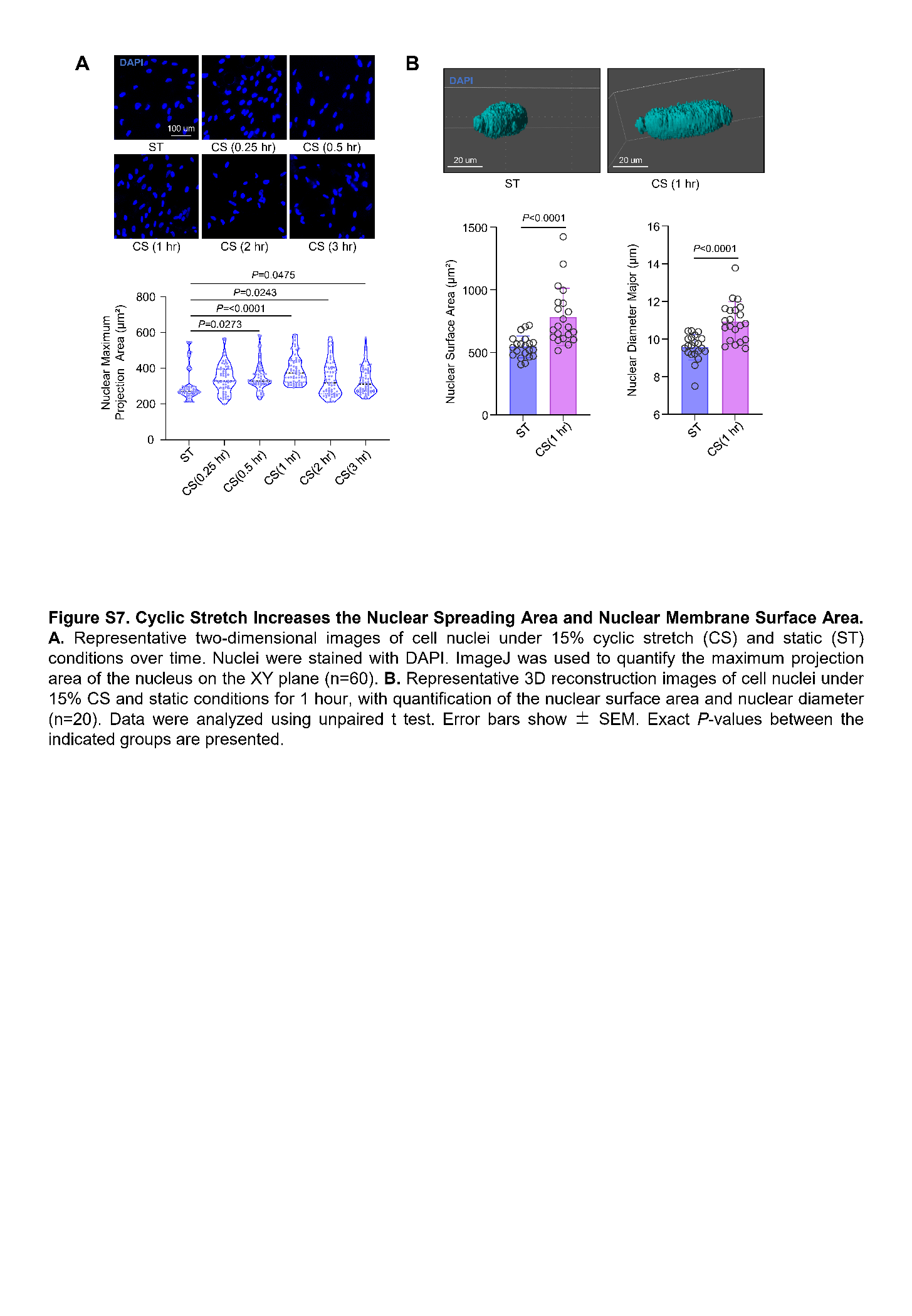


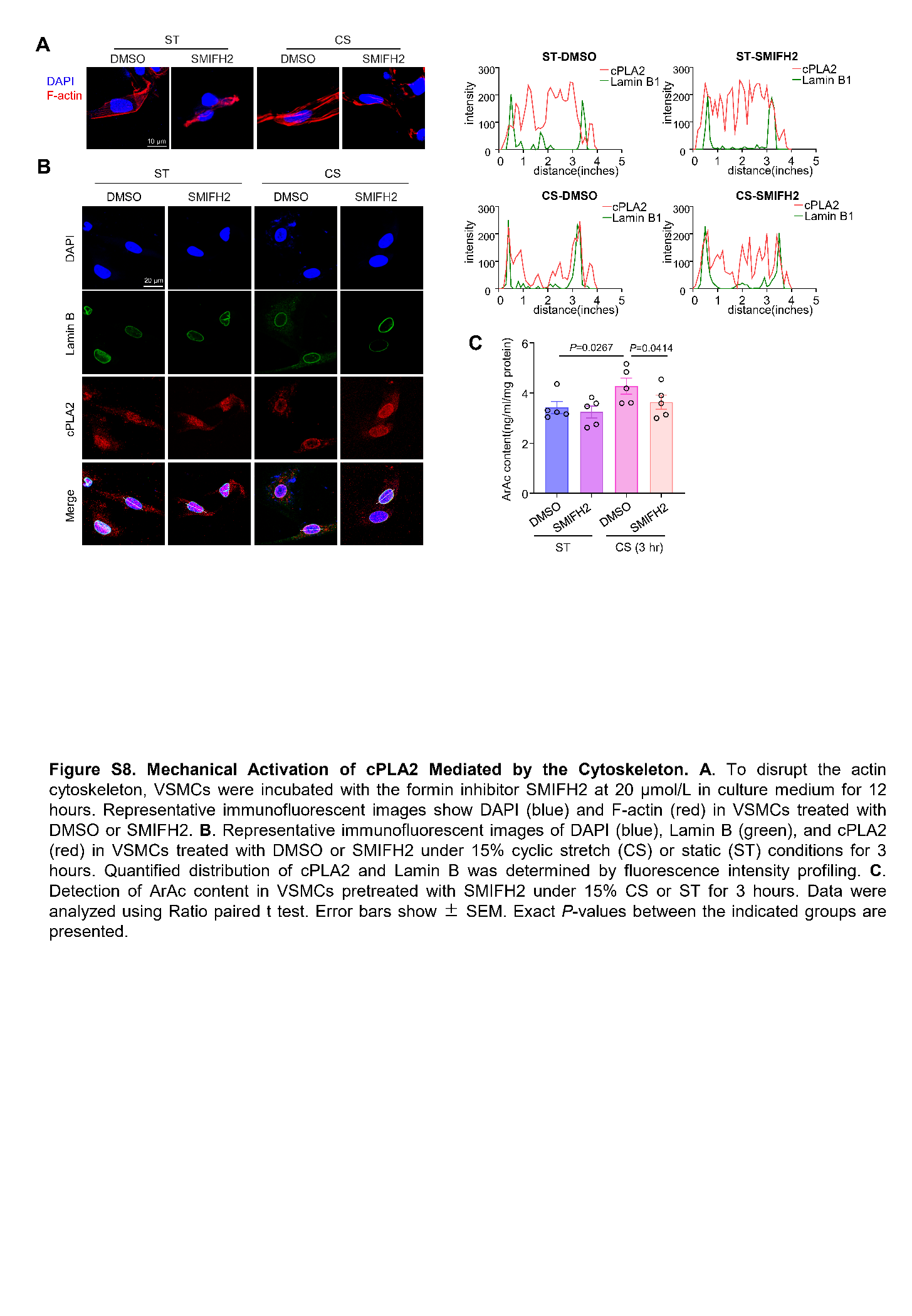


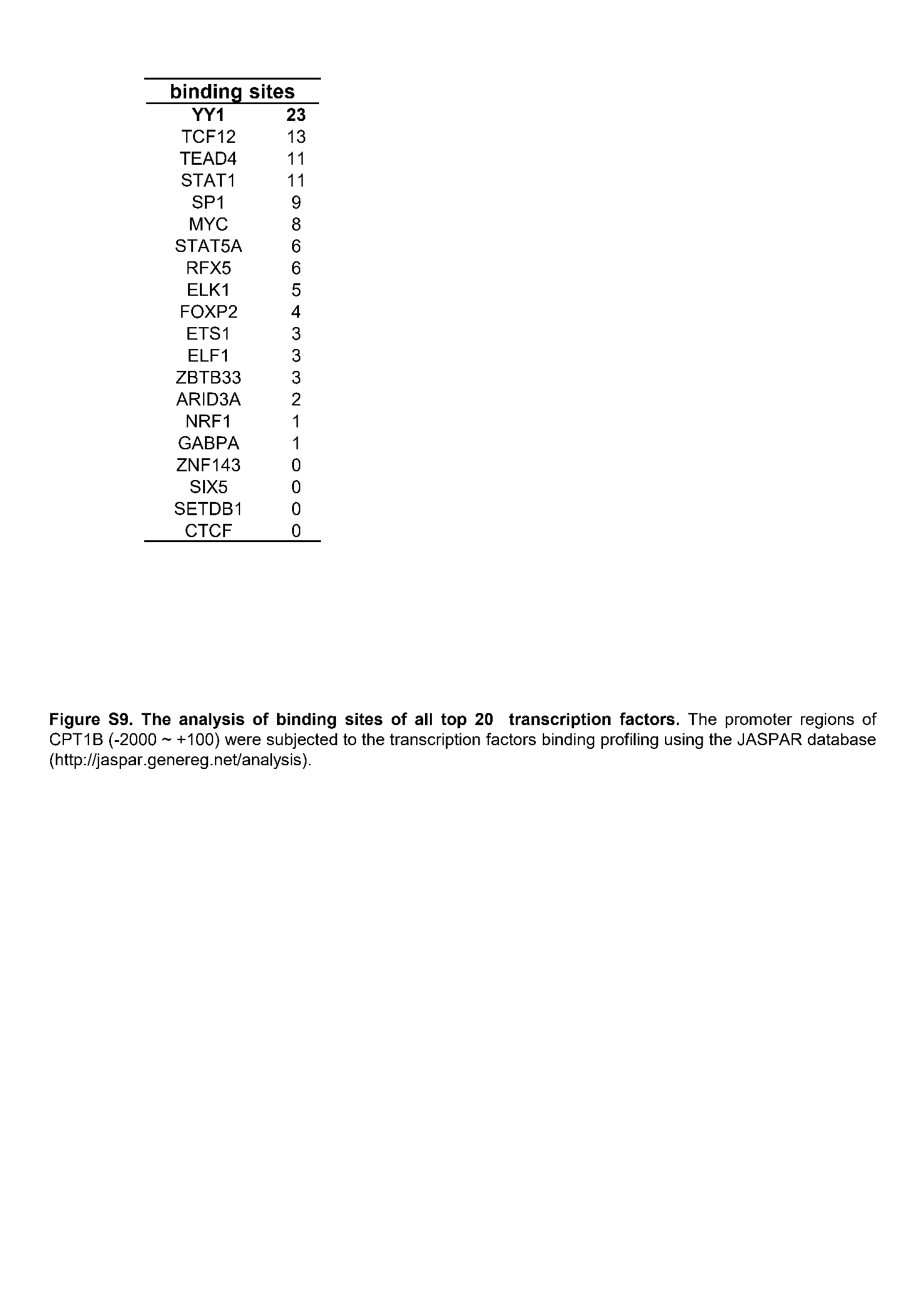


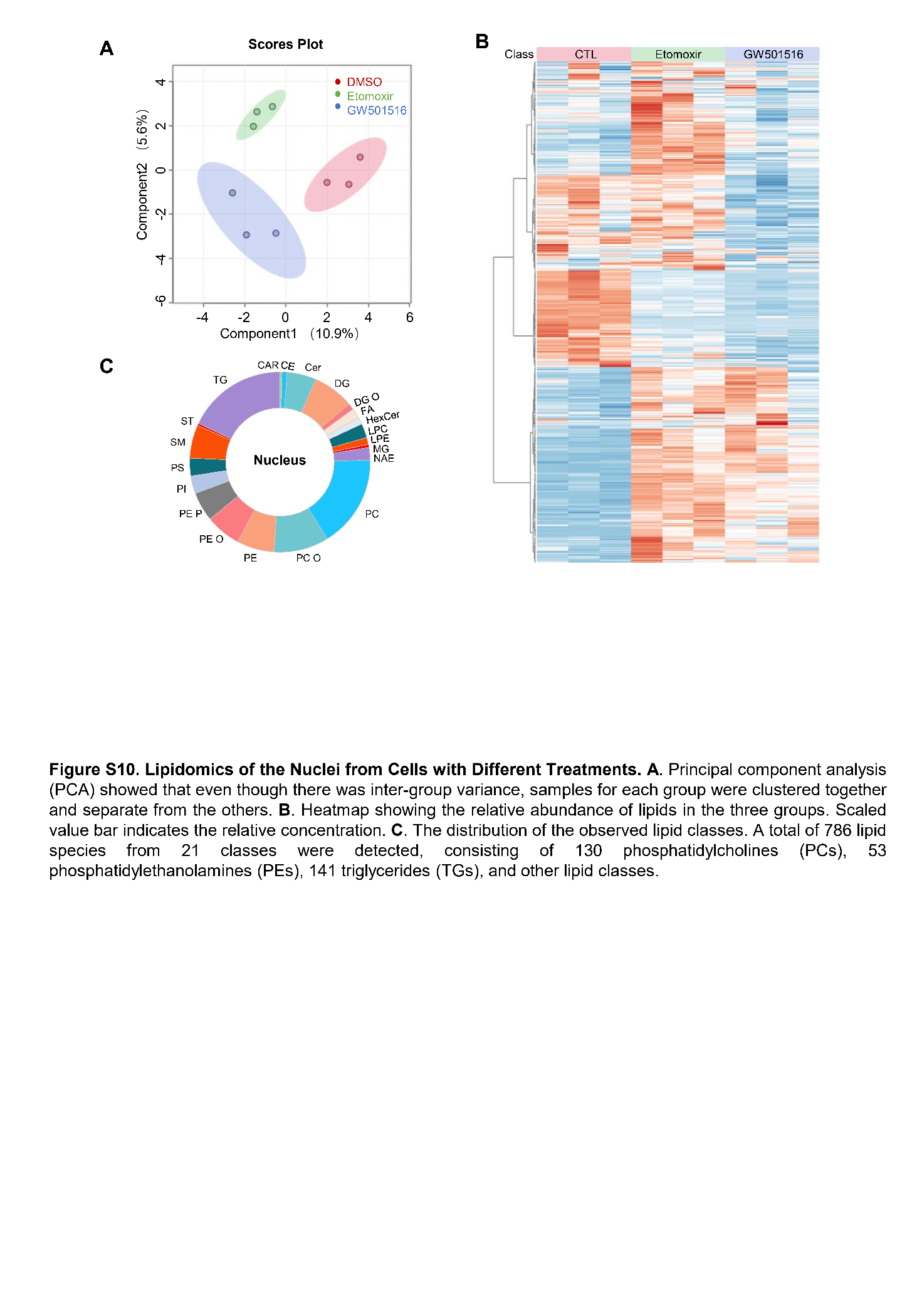


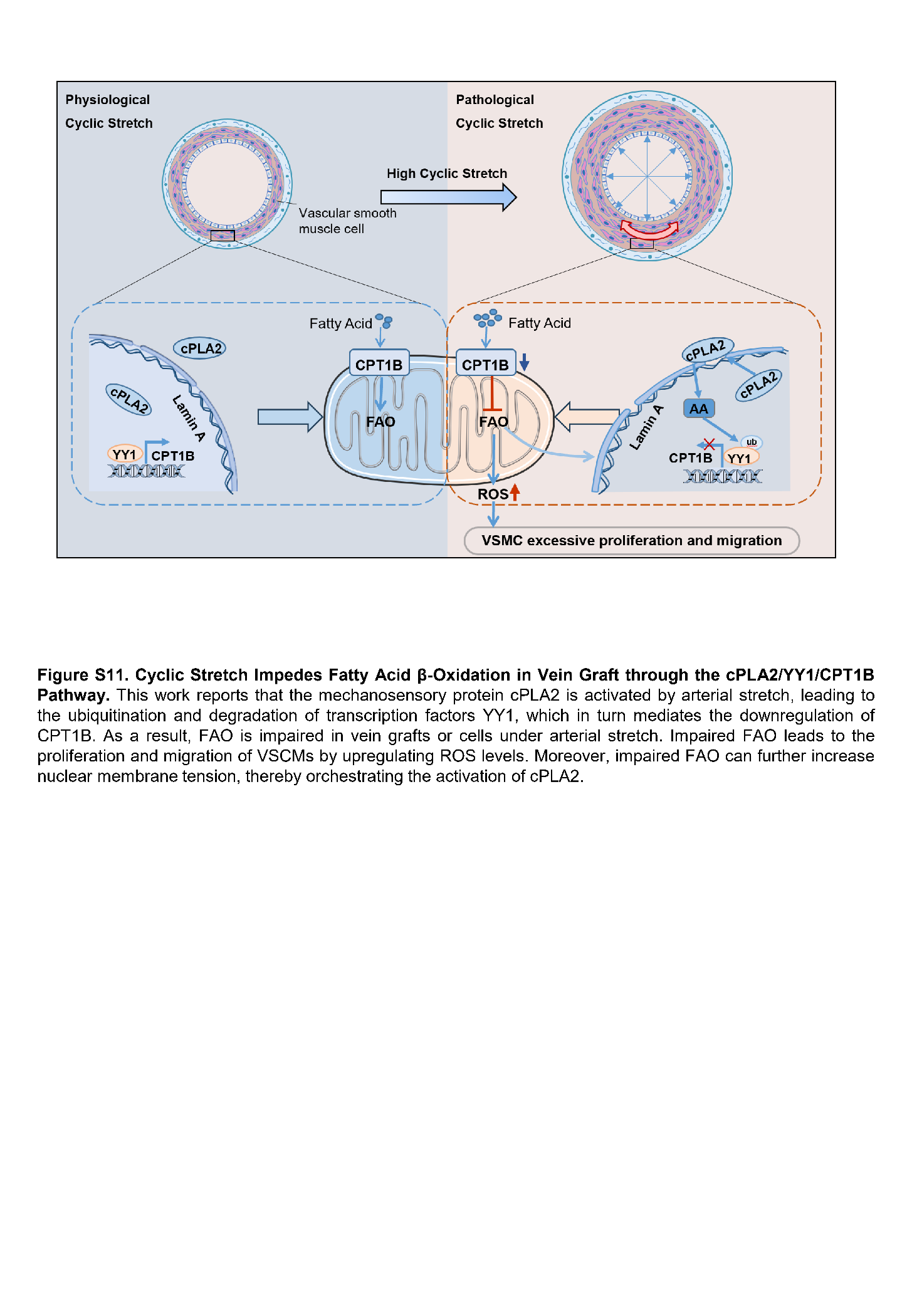

Supplement: Supplementary file 1 — Supporting Information [file ADVS-12-2411559-s001.docx]
